# k-OptForce: Integrating Kinetics with Flux Balance Analysis for Strain Design

**DOI:** 10.1371/journal.pcbi.1003487

**Published:** 2014-02-20

**Authors:** Anupam Chowdhury, Ali R. Zomorrodi, Costas D. Maranas

**Affiliations:** Department of Chemical Engineering, Pennsylvania State University, University Park, Pennsylvania, United States of America; University of Michigan, United States of America

## Abstract

Computational strain design protocols aim at the system-wide identification of intervention strategies for the enhanced production of biochemicals in microorganisms. Existing approaches relying solely on stoichiometry and rudimentary constraint-based regulation overlook the effects of metabolite concentrations and substrate-level enzyme regulation while identifying metabolic interventions. In this paper, we introduce k-OptForce, which integrates the available kinetic descriptions of metabolic steps with stoichiometric models to sharpen the prediction of intervention strategies for improving the bio-production of a chemical of interest. It enables identification of a minimal set of interventions comprised of both enzymatic parameter changes (for reactions with available kinetics) and reaction flux changes (for reactions with only stoichiometric information). Application of k-OptForce to the overproduction of L-serine in *E. coli* and triacetic acid lactone (TAL) in *S. cerevisiae* revealed that the identified interventions tend to cause less dramatic rearrangements of the flux distribution so as not to violate concentration bounds. In some cases the incorporation of kinetic information leads to the need for additional interventions as kinetic expressions render stoichiometry-only derived interventions infeasible by violating concentration bounds, whereas in other cases the kinetic expressions impart flux changes that favor the overproduction of the target product thereby requiring fewer direct interventions. A sensitivity analysis on metabolite concentrations shows that the required number of interventions can be significantly affected by changing the imposed bounds on metabolite concentrations. Furthermore, k-OptForce was capable of finding non-intuitive interventions aiming at alleviating the substrate-level inhibition of key enzymes in order to enhance the flux towards the product of interest, which cannot be captured by stoichiometry-alone analysis. This study paves the way for the integrated analysis of kinetic and stoichiometric models and enables elucidating system-wide metabolic interventions while capturing regulatory and kinetic effects.

## Author Summary

Computational strain design procedures aim at assisting metabolic engineering efforts by identifying metabolic interventions leading to the targeted overproduction of a desired chemical using network models of cellular metabolism. The effect of metabolite concentrations and substrate-level enzyme regulation cannot be captured with stoichiometry-only metabolic models and analysis methods. Here, we introduce k-OptForce, an optimization-based strain design framework incorporating the mechanistic details afforded by kinetic models, whenever available, into a genome-scale stoichiometric-based modeling formalism. The resulting optimization problems pose significant computational challenges due to the bilevel nature of the formulation and the nonconvex terms in the constraints. A tractable reformulation and solution procedure is introduced for solving the optimization problems. k-OptForce uses kinetic information to (re)apportion reaction fluxes in the network by identifying interventions comprised of both direct enzymatic parameter changes (for reactions with available kinetics) and reaction flux changes (for reactions with only stoichiometric information). Our results show that the introduction of kinetic expressions can significantly alter the identified interventions compared to those identified with stoichiometry-alone analysis. In particular, additional modifications are required in some cases to avoid the violation of metabolite concentration bounds, while in other cases, the kinetic constraints yield metabolic flux distributions that favor the overproduction of the desired product thereby requiring fewer direct interventions.

## Introduction

Bio-production is emerging as a competitive strategy for the production of a wide range of chemicals ranging from biofuels, precursor chemicals and bioactive molecules (see [1]–[3] for detailed reviews). The use of metabolic modeling and computations is increasingly becoming instrumental in deciding how to engineer the production strain [4]–[11]. Computational strain design generally involves solving an optimization problem which optimizes a specific performance requirement (e.g., maximum flux of desired product) while minimizing the total number of genetic alterations in the metabolic model. Depending on the adopted description of metabolism strain design computational tools could be broadly categorized as based on stoichiometry-alone or kinetic models of metabolism [12].

Kinetic models of metabolism require quantitative expressions that link reaction fluxes and metabolite concentrations. A system of ordinary differential equations (ODEs) is typically solved to obtain the time-dependent variation in metabolite concentrations and reaction fluxes. Different forms of mechanistic expressions have been used extensively such as Michaelis-Menten or Hill Kinetic expressions [13], [14]. These expressions require *a priori* knowledge of detailed enzyme function mechanism and characterization [15], [16]. Alternatively, various approximate kinetic forms such as lin-log [17]–[19] and log-lin [20] kinetics, power law kinetic expressions such as the S-system [21] and Generalized Mass Action [22], and other forms of cooperativity and saturation [23], [24] and convenience rate laws [25] have been proposed to reduce the number of kinetic parameters and complexity of the rate expressions. In addition, Varner and Ramkrishna [26]–[28] pursued the development of kinetic descriptions inspired by cybernetic modeling and optimality concepts. A number of review articles highlight the merits and demerits of various kinetic modeling formalisms [17], [29], [30]. Uncertainty in the assignment of kinetic parameter values has motivated the development of approaches that do not fix the parameter values but rather sample from a probability distribution [31]–[34]. Even though the use of kinetic models have led to some successes for strain design [20],[35]–[43] the relative small scope of the employed models, difficulties in obtaining kinetic expressions and questionable portability of kinetic expressions across microbial production platforms have so far limited wide applicability and acceptance.

The introduction of genome-scale models of metabolism [44]–[46] and the use of Flux Balance Analysis (FBA) to assess their maximum theoretical yields [47], flux ranges [48] and trade-offs between growth and productivity [49] led to a flurry of computational strain design approaches [50], [51] that used a purely stoichiometric description of metabolism. The advantage of using stoichiometry alone supplemented with some regulatory information is that the widest possible range of potentially feasible metabolic phenotypes could be accessed. The linearity of the underlying FBA description also affords significant computational savings and tractability even for genome-scale models. The downside is that identified flux redirection predictions (especially knock up/downs) are sometimes hard to translate into an actionable genetic intervention. For example, it is unclear if a desired metabolic flux up-regulation is achievable or even consistent with enzyme kinetics and/or whether it may lead to physiologically problematic metabolite concentrations.

Stoichiometry-based strain design algorithms are often structured as bilevel mixed integer linear optimization problems (MILP) [4], [8], [9], [11], [52]. The outer level optimizes the biotechnological objective (i.e. overproduction of target chemical) through metabolic interventions, while the inner level optimizes the cellular objective that tries to counteract any external imposed genetic or environmental modifications [53], [54]. Different fitness functions have been identified to simulate the cellular objective including maximization of biomass yield [4], [8], minimization of metabolic adjustment [5], [54], regulatory on-off minimization [55], [56], worst-case scenario [9], [52], or a combination thereof [11], [57], [58]. Details of these procedures have been reviewed elsewhere [50], [51]. Even though they may lack important information on the enzyme kinetics of reactions these procedures have been successfully employed for the strain design of many important chemical products [59]–[64].

The need to integrate the mechanistic detail (whenever available) of kinetic expressions with the genome-scale scope of stoichiometric models has been recognized early by the community. Dynamic Flux Balance Analysis (dFBA) [65] integrates uptake kinetic expressions of the carbon substrate while optimizing biomass at every time step to apportion fluxes to the rest of the metabolic network. Several other researchers [66]–[68] extended this approach to incorporate kinetic expressions of multiple carbon sources and other nutrients into their quasi steady-state formalisms. Zhuang *et al* [69] and Salimi *et al* [70] developed the dynamic multi-species metabolic modeling (DMMM) approach to incorporate uptake kinetics of metabolites in stoichiometric models of a microbial consortium. Alternatively, steady-state flux distributions (from FBA) and stoichiometric information have been used to parameterize genome-scale kinetic models valid for small perturbations [22], [71]–[73]. For example, Fleming *et al* [74] incorporated lin-log kinetic expressions from a small *E. coli* model (comprised of 76 equations) to constrain an FBA simulation. Similarly, Cotton *et al* [75] performed Flux Variability Analysis (FVA) for each flux in a small kinetic model (by allowing the kinetic parameters to vary about their steady-state values) and used the tighter bounds on kinetic parameters to refine flux estimation in genome-scale models. Despite these advancements on the modeling front, the use of hybrid stoichiometric/kinetic models has been left largely unexplored in the context of strain design.

Here, we introduce k-OptForce, which extends the previously developed OptForce procedure [9] by bridging this gap between stoichiometry-only and kinetics-based descriptions of metabolism. This procedure seamlessly integrates the mechanistic detail afforded by kinetic models within a constraint-based optimization framework tractable for genome-scale models. Instead of relying on surrogate fitness functions such as biomass maximization or worst-case simulation for predicting flux re-directions, k-OptForce uses kinetic rate expressions to (re)apportion fluxes in the metabolic network. Using mechanistic models available in literature (for example kinetic models for the central metabolism of *E. coli* [13], [76], [77] and *S. cerevisiae* [14], [78], [79]) the allowable phenotype of both the reference and the engineered strain are characterized to be consistent with the allowable kinetic space. Subsequently, alternative genetic intervention strategies consistent with the restrictions imposed by maximum enzyme activity and kinetic regulations, as well as with the worst-case scenario of production of the desired chemical are identified using a bilevel optimization framework. We benchmarked the k-OptForce protocol for the microbial overproduction of L-serine in *E. coli*, and triacetic acid lactone (TAL) in *S. cerevisiae*. For the former, k-OptForce identified key regulatory bottlenecks in upper and lower glycolysis that must be overcome to redirect more flux towards L-serine, which regular OptForce fails to pinpoint. In addition, k-OptForce removed interventions identified by regular OptForce that resulted in kinetically infeasible flux re-distributions. Application of the k-OptForce for the microbial overproduction of TAL in *S. cerevisiae* revealed the impact of additional kinetic constraints in alleviating a severe worst-case simulation of regular OptForce, resulting in a higher prediction of TAL yield from fewer interventions as compared to regular OptForce predictions. The introduction of kinetic expressions in strain design can significantly affect the identified interventions in sometimes non-intuitive ways. In some cases additional modifications are needed to substitute interventions that cause enzyme saturation or concentration bound violations. The mechanism of action of these modifications is often subtle by alleviating substrate inhibition or draining away cofactors from competing pathways. In other cases, kinetic expressions shape flux distributions so as to favor the overproduction of the desired product requiring fewer direct interventions.

## Methods

**Figure 1:**
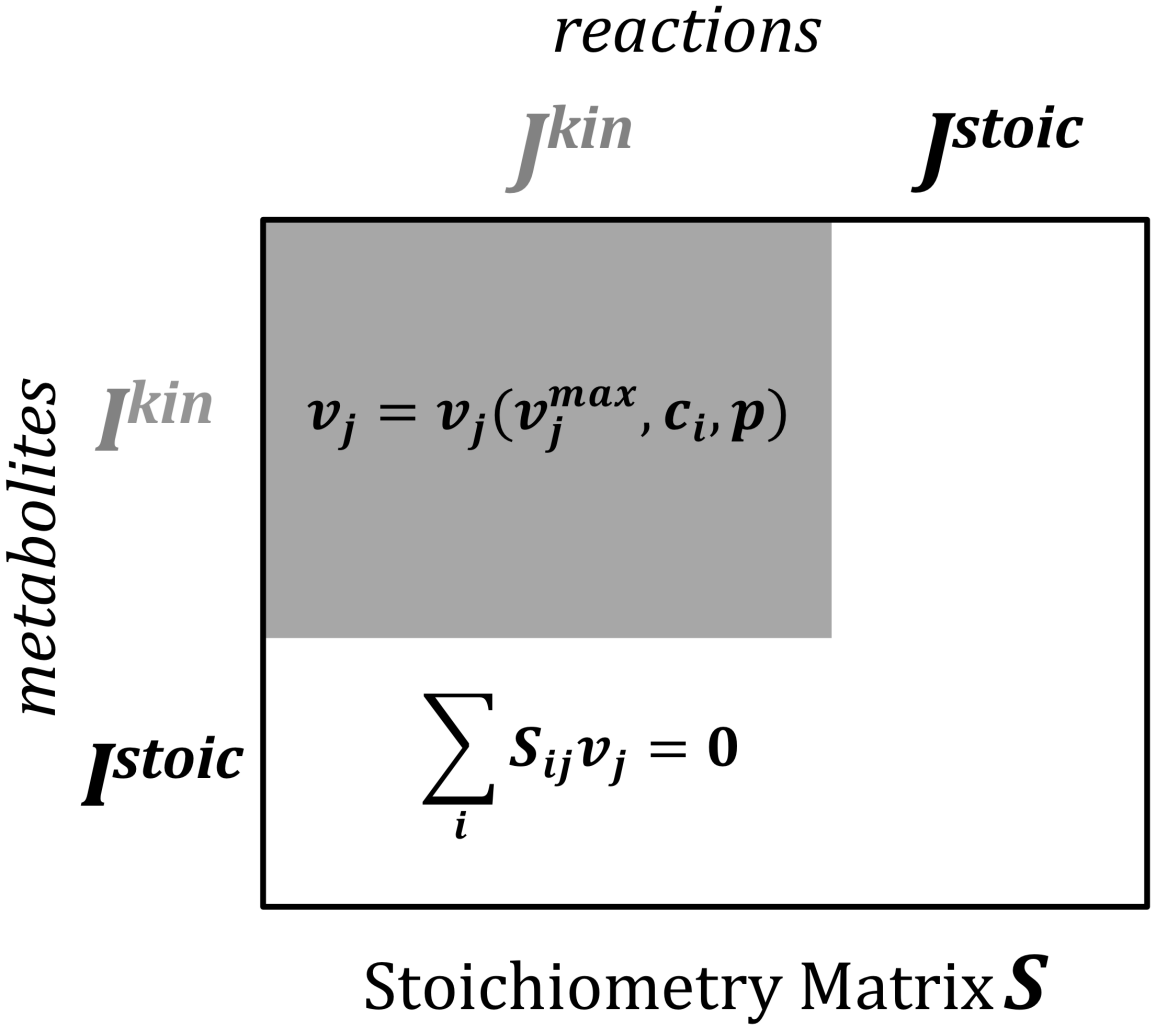
Schematic diagram showing the partition of reactions into the ones with kinetic information *J^kin^* and those linked by only stoichiometry *J^stoic^*. Note that some metabolites participate in only the stoichiometric part of the model (i.e., ***I^stoic^***) whereas others participate in both (i.e., ***I^kin^***). The flux of reaction *j* (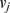) in the kinetic part of the model (shaded region) is determined by the kinetic formalism, 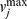, metabolite concentrations 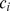 and other kinetic parameters *p* while the ones in ***J^stoic^*** by the mass conservation laws (non-shaded region).

k-OptForce aims at utilizing the available kinetic information within a larger genome-scale stoichiometric model to more accurately characterize all possible metabolic phenotypes of the reference and engineered strains. The procedure builds upon the OptForce procedure [9] by augmenting the metabolic network description with kinetic rate laws whenever available. The reactions in the metabolic network are partitioned into two subsets: reactions with kinetic information ***J^kin^*** = {*j*|*j* = 1,2,…,*N^kin^*} and reactions with only stoichiometric information ***J^stoic^*** = {*j*|*j* = 1,2,…,*N^stoic^*} (see Figure 1). Metabolic flux of reactions in ***J^stoic^*** is constrained only by stoichiometric balances and reaction directionality restrictions whereas flux for reactions in ***J^kin^*** is fully determined by enzyme activity, metabolite concentrations and kinetic parameter values. The ***J^kin^*** part of the metabolic network is mathematically described by a system of (usually) nonlinear ordinary differential equations (ODEs) denoting the non-steady-state balance for each metabolite. For a square system of ODEs integration yields steady-state metabolite concentrations and fluxes. The allowable metabolic phenotype (i.e., flux ranges) of the remaining portion of the network ***J^stoic^*** is inferred so as to be consistent with the predicted fluxes of the reactions in ***J^kin^***.

The k-OptForce procedure is composed of the following steps:

### Characterization of the reference (e.g., wild-type) strain

**Figure 2:**
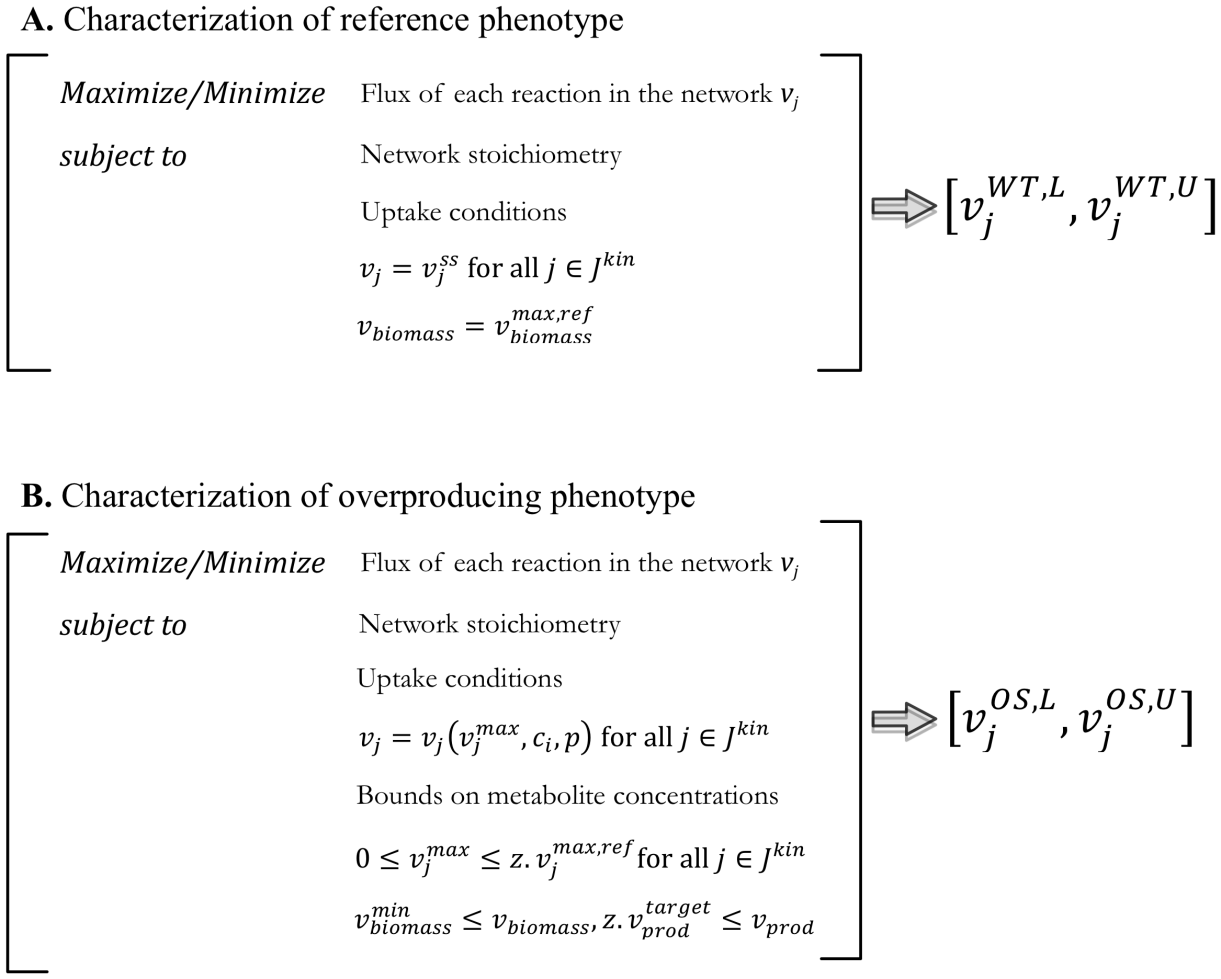
Optimization structure for the characterization of the reference and the phenotype consistent with overproduction of target chemical. (A) The reference phenotype characterization identifies the minimum 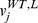 and maximum 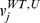 flux limits of all reactions consistent with the steady-state reference flux 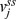 of the reactions in ***J^kin^*** and the maximum biomass production 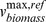. (B) The overproducing phenotype is identified by calculating the minimum 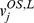 and maximum 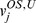 flux limits of all reactions consistent with the kinetic expressions for ***J^kin^***, the minimum target production of desired chemical 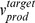 and biomass 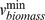. The metabolite concentrations and enzyme activities are allowed to vary within a pre-specified range of their reference values.

The system of ODEs is solved first to obtain steady-state fluxes for reactions in ***J^kin^***. The phenotypic space of the reference strain is then identified by iteratively maximizing and minimizing the flux of each reaction in ***J^stoic^*** while keeping the fluxes of reactions in ***J^kin^*** fixed at their steady-state values, and restricting the flux of any other reaction for which any experimental data is available at their experimentally determined values or ranges (Figure 2A and Supplementary Text S1).

### Characterization of the overproducing strain

The flux ranges of the strain consistent with a desired overproduction target are similarly constructed by successively maximizing and minimizing the flux of network reactions subject to network stoichiometry, overproduction target, while also incorporating the kinetic expressions for reactions in ***J^kin^*** as additional constraints. The resulting optimization formulation is shown qualitatively in Figure 2B (see Supplementary Text S1 for detailed formulation). Up/down flux regulations within ***J^kin^*** are modeled by modulating the corresponding maximum enzymatic reaction rates 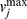 using a scalar ***z*** to denote the maximum allowable departure from their reference values 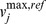. A value for z of zero denotes a knock-out, a value less one implies a down-regulation whereas a value greater than one an up-regulation. The metabolite concentration ranges involved in the kinetic expressions are selected by selecting a percent allowed departure from the values obtained by solving the system of ODEs (e.g., +/−50%) and/or experimentally derived measurements. Nonlinear kinetic formalisms give rise to nonconvex nonlinear optimization problems (NLP) for identifying the flux ranges of the overproducing strain. These problems are solved to optimality using the global optimization solver BARON [80] accessed through GAMS. It is important to note that the kinetic expressions significantly restrict the range of allowable flux values consistent with experimental data and/or an overproduction target compared to flux ranges constrained only by stoichiometry. We quantify the average extent of this contraction by defining the Average Bound Contraction (ABC) factor:
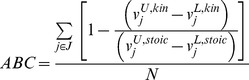
Here 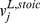 and 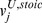 denote the respective lower and upper bounds for the fluxes consistent with the overproduction target when only stoichiometry is used and 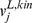 and 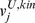 represent the same bounds upon the incorporation of kinetic information. Interestingly, kinetic information for reactions in ***J^kin^*** propagates into ***J^stoic^*** leading to bound contraction even for reactions with stoichiometry-only description. Tighter description of allowable flux ranges for the reference and overproducing strains allow for sharper elucidation of reactions whose flux must change to meet the imposed target (i.e., MUST sets).

### Identification of MUST sets

Similar to the OptForce procedure [9], by contrasting the flux space of the wild-type network with that of the overproducing strain, the sets of reactions that must be up-regulated (***MUST^U^***), down-regulated (***MUST^L^***), or be knocked out ***(MUST^X^)*** are identified (see supplementary text of Ranganathan *et al* [9] for details). This procedure could be extended to identify higher order MUST sets (e.g., MUST Doubles, Triples etc.) where instead of comparing the flux ranges for individual reactions, we contrast the sum and/or difference of two or more fluxes (depending on the order) between the reference and the desired phenotypes. For example, this procedure can elucidate ***MUST^UU^***, ***MUST^LL^*** and ***MUST^UL^*** sets (see supplementary text of Ranganathan *et al* [9] for details).

### Identification of FORCE sets

FORCE set is the minimum set of reactions (and by extension genetic) manipulations selected from within the MUST sets whose direct manipulation (i.e., updating of lower or upper bounds) ensures production of the desired chemical beyond a desired target even under the worst-case scenario where fluxes are re-apportioned to drain flux away from the target product.

**Figure 3:**
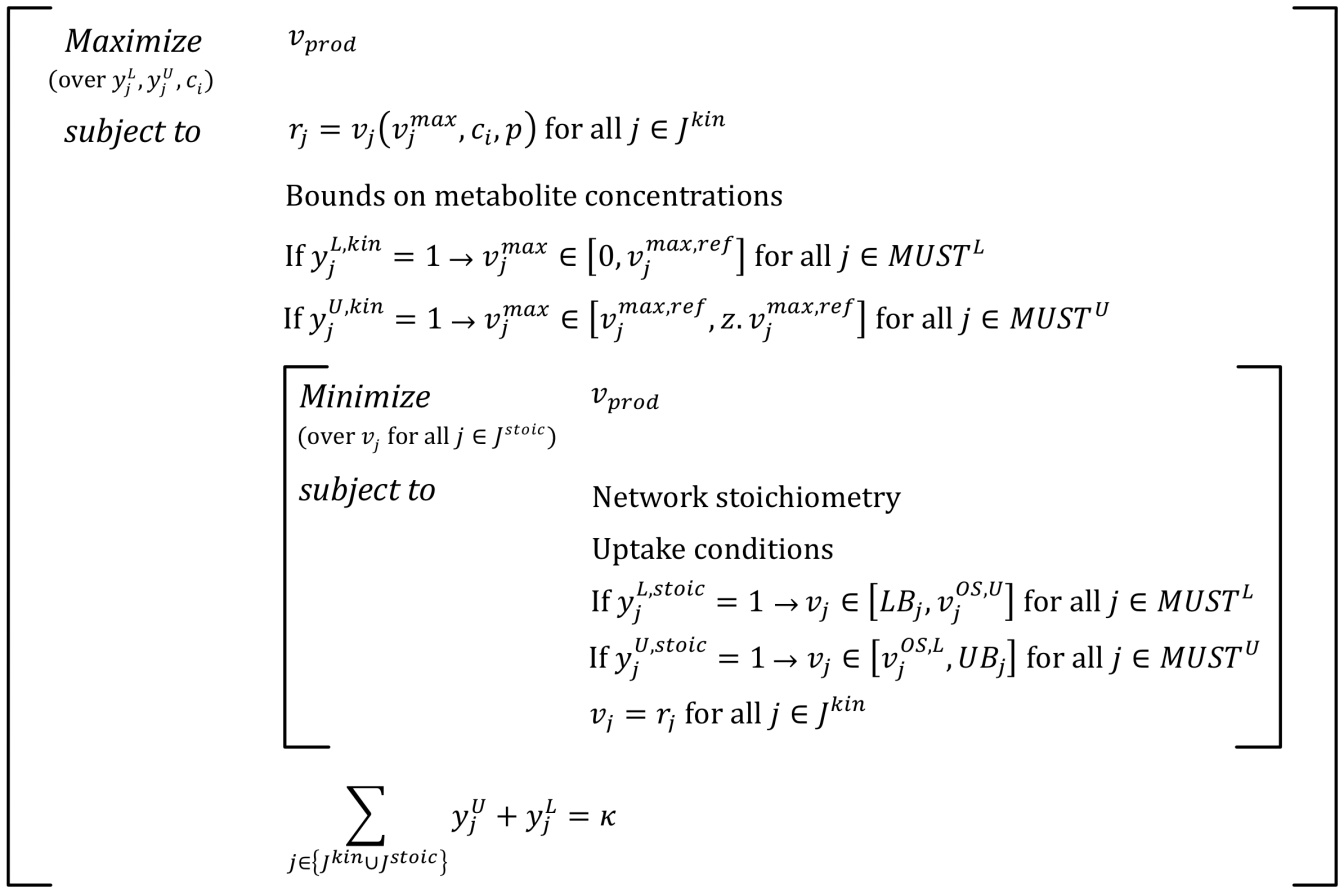
Single-step bilevel formulation for the identification of FORCE sets. The outer problem maximizes the flux towards the desired chemical while the inner problem simulates the worst-case scenario by minimizing the product flux. Binary variables 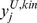 and 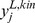 propagate the effect of engineering modifications in ***J^kin^*** while 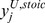 and 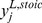 do the same in ***J^stoic^***. The fluxes in ***J^kin^*** remain unchanged in the inner problem.

The worst-case scenario is mathematically described by extending the bilevel optimization problem used for original OptForce [9], as shown in Figure 3 (also see Supplementary Text S1 for the detailed procedure). The outer problem maximizes the flux towards the desired chemical consistent with reaction kinetics and stoichiometry. Binary variables *y^L,kin^* and *y^U,kin^* associated with the ***MUST^L^*** and ***MUST^U^*** sets of reactions in ***J^kin^*** respectively, are used to control the effect of engineering modifications to the corresponding 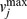. If *y^L,kin^* = 1 then 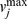 for that reaction can be down-regulated to a value between 0 and its wild-type 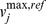. If *y^U,kin^* = 1 then 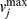 for that reaction can be up-regulated to a value between 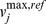 and 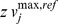. Otherwise, 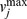 is kept unaffected at the reference 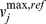 value. As in the original OptForce procedure, the inner problem simulates the worst-case scenario by minimizing product formation but only for the reactions in ***J^stoic^***. A separate set of binary variables *y^L,stoic^* and *y^U,stoic^* identify interventions in ***J^stoic^*** required to guarantee a non-zero yield of the target chemical consistent with the flux distribution in ***J^kin^***. It is important to note that the metabolic fluxes in ***J^kin^*** remain unaffected by the worst-case simulation of the reactions in ***J^stoic^*** in the inner problem.

This bilevel formulation is converted into a single level mixed integer nonlinear problem (MINLP) using the conditions of strong duality. We construct the dual of the inner problem (called the primal), and add all the dual constraints, along with those of the primal, to the outer optimization problem. Since all the nonlinear kinetic expressions are present in the outer problem, the inner (primal) problem is linear in the reaction fluxes *v_j_*. The fitness function of the inner problem is imposed setting the objectives of the primal and the dual equal to each other. It is to be noted here that the dualization of the flux variable for each reaction in ***J^kin^*** introduces a bilinear term to the single-level formulation. This can be avoided by imposing the Karush-Kuhn-Tucker (KKT) conditions of complementary slackness between the primal constraints and their dual variables. This leads to the introduction of a binary variable for each constraint in the primal problem which is generally more tractable than the original bilinear constraints (see Supplementary Text S1 for detailed formulations). The above described sequence of equivalent problem re-formulations yields a single-level nonlinear MINLP (k-OptForce). The single-level k-OptForce optimization is successively solved using the global solver BARON [80] for an increasing number of interventions (by increasing 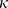) until the target yield is met.

**Figure 4:**
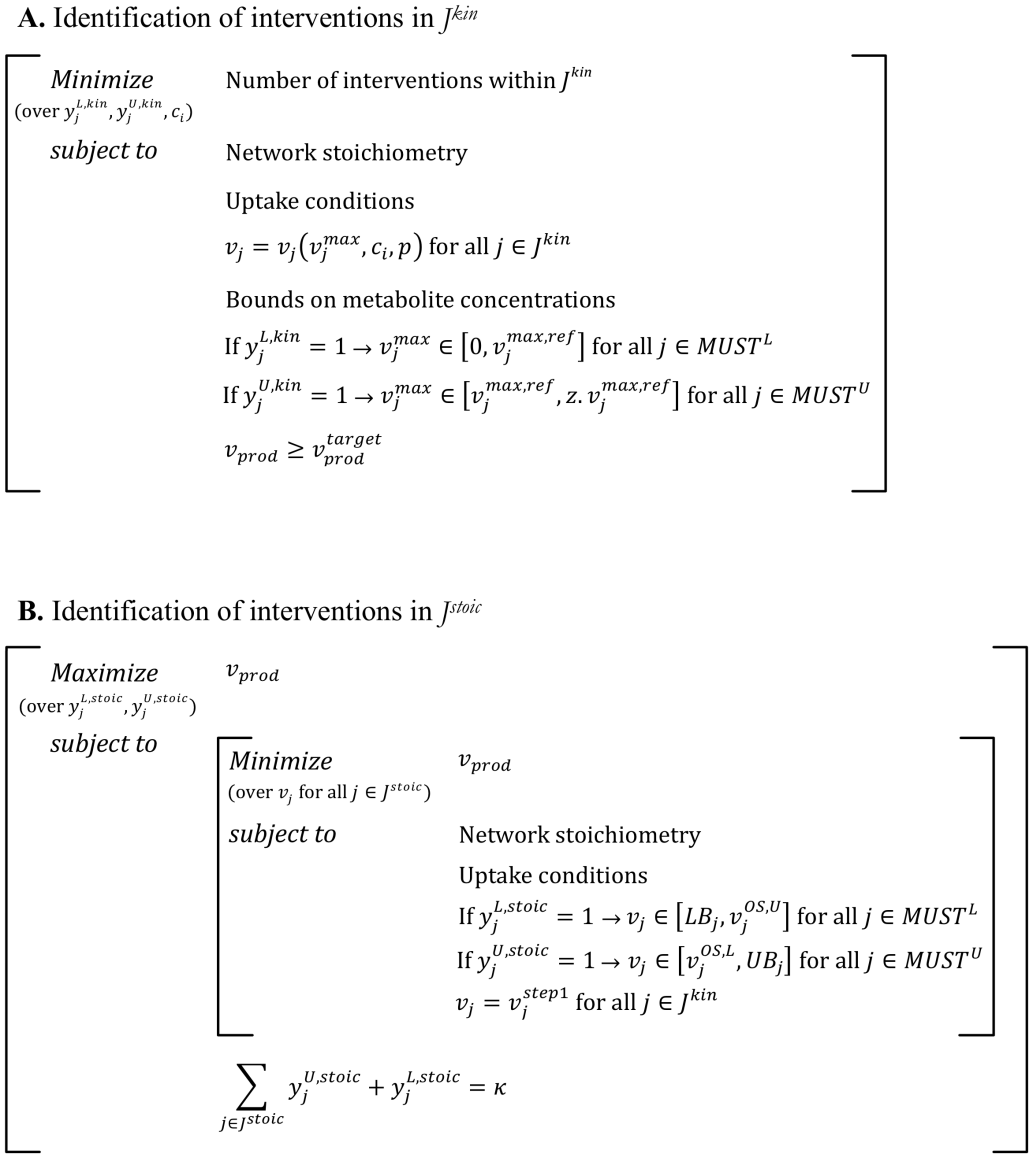
Two-step formulation for the identification of the FORCE sets. (A) The first step of the formulation identifies the minimum number of interventions (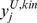 and 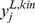) in ***J^kin^*** required to meet the desired levels of overproduction of target chemical. (B) The second step identifies the additional interventions (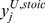 and 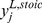) in ***J^stoic^*** that would guarantee the desired yield in the worst case scenario.

Due to the nonconvex nature of the kinetic expressions and the large number of binary variables the resulting MINLP equivalent representation of the bilevel optimization problem may become computationally intractable. For these cases, we exploit the natural hierarchy of the model by first selecting interventions within ***J^kin^*** and subsequently within ***J^stoic^*** (see Figure 4 and Supplementary Text S1 for the algorithmic details). In the first step, a nonconvex optimization problem is solved to identify the minimum number of manipulations in ***J^kin^*** that are consistent with the overproduction target (Figure 4A). Keeping the fluxes in ***J^kin^*** fixed at their optimized values the flux ranges of the overproducing strain are then re-calculated and the FORCE set for reactions in ***J^stoic^*** are then identified (see Step 2) as in regular OptForce [9] (Figure 4B). By solving two separate problems the computational burden is significantly reduced at the expense of potentially missing synergistic interventions that share reactions between ***J^kin^*** and ***J^stoic^***. It is to be noted that while the illustrated formulation only targets changes in 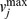 for reactions in ***J^kin^*** the same analysis could be applied for the modulation of other kinetic parameters (e.g., *K_m_*, *K_I_* etc.) in the model.

Once the FORCE set of interventions are identified (along with alternative manipulation strategies), it is important to manually curate the results to understand the underlying metabolic reason behind each intervention. This is necessary since k-OptForce makes use of not just stoichiometry which imposes straightforward connections between reactants and products but also kinetics that introduce complex nonlinear couplings often between distal reactions through metabolite pools. While it is not possible to put forth an invariant set of rules for all applications, the following checks can be useful in general: (1) check whether any metabolite participating in affected reactions is hitting lower or upper concentration bounds, (2) identify if a common metabolite is fixing the branching ratio of fluxes in two pathways, (3) resolve whether a metabolite is limiting the flux of a reaction through substrate-level inhibition, (4) confirm if the flux in a pathway has been restricted because the *v^max^* of one of the reactions has hit its upper bound, and, (5) analyze all alternate intervention strategies to identify common metabolites and/or enzymes that are being targeted.

## Results

We contrasted k-OptForce with the original OptForce [9] predictions for the overproduction of L-serine and TAL in *E. coli* and *S. cerevisiae*, respectively. The goal was to assess how the addition of kinetic information into stoichiometric models changes the list of identified interventions and more importantly what are the underlying reasons for the new interventions.

### Overproduction of L-serine in *E. coli*

**Figure 5:**
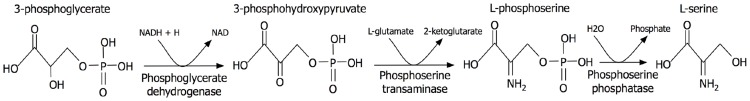
Pathway for L-serine production in *E. coli*.

L-Serine is a non-essential amino acid and a precursor for other amino acids such as cysteine, tryptophan and glycine. It also participates in the biosynthesis of purines and pyrimidines, and serves as an intermediate for phospholipids, sphingolipids and folate synthesis for several microorganisms [81], [82]. The synthesis of L-serine in microorganisms such as *Escherichia coli* and *Corynbacterium* consists of a three-step pathway branching out of the glycolytic intermediate 3-phosphoglycerate (3pg). 3pg is converted to 3-phosphohydroxypyruvate (3php) by phosphoglycerate dehydrogenase (PGCD, EC 1.1.1.95), and phosphoserine transaminase (PSERT, EC 2.6.1.52) catalyzes the conversion of 3-phosphohydroxypyruvate (3php) to L-phosphoserine (pser-L) using L-glutamate as the amino acid donor. In the last step, phosphoserine phosphatase (PSP, EC# 3.1.3.3) catalyzes the final conversion to L-serine (see Figure 5). We used the genome-scale *i*AF1260 model of *E. coli* [83] as the stoichiometric model for our simulations. The kinetic rate expressions for reactions of central metabolism were extracted from Chassagnole *et al* [76]. This kinetic model, which has been used before in variety of studies [5], [36], [39], [84], consists of 25 metabolites and 25 reactions from glycolysis and pentose phosphate pathway (see Supplementary Material S1). All simulations were carried out in aerobic minimal medium with glucose as the sole carbon source.

The reference strain (i.e., wild-type *E. coli*) flux ranges were identified by finding the maximum flux variability in the entire network while keeping the fluxes in ***J^kin^*** fixed at the steady-state values obtained by solving the system of ODEs for the kinetic model (see Supplementary Figure S1). The L-serine overproducing network flux ranges were calculated for a target of 90% maximum theoretical yield (i.e., 180 mol/100 mol glucose uptake). The minimum biomass production was kept at 10% of its maximum achievable. The maximum enzyme activity *v^max^* of reactions in ***J^kin^*** was allowed to vary from zero to two-fold up-regulation of its reference activity (i.e., z = 2). Also, the concentration of metabolites in ***I^kin^*** was allowed to vary within a two-fold range from their steady-state concentrations in the reference strain.

**Figure 6:**
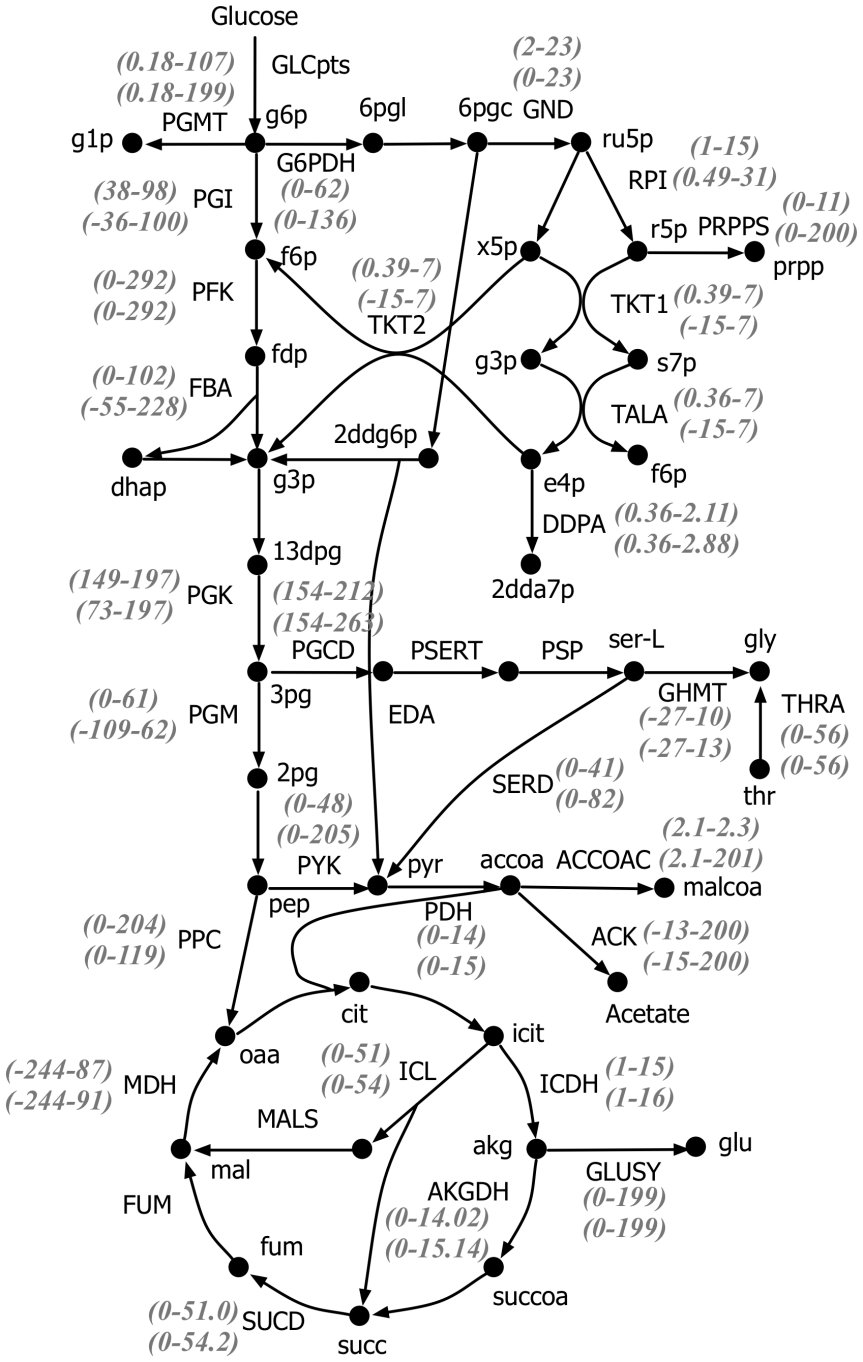
Reduction in the flux ranges of the desired phenotype for L-serine overproduction in *E. coli* after the incorporation of additional kinetic constraints. Values on top indicate reduced flux ranges (in mmol gDW^−1^ hr^−1^), while values in the bottom indicate the flux ranges (in mmol gDW^−1^ hr^−1^) without any kinetic information, for 100 mmol gDW^−1^ hr^−1^ of glucose uptake.

Figure 6 illustrates the reduction in flux ranges in the overproducing phenotype after the introduction of the kinetic constraints when compared with a stoichiometry-only description. The average bound contraction (ABC) was 52% for the fluxes in ***J^kin^***. For example, the flux of glucose 6-phosphate dehydrogenase (G6PDH) in oxidative pentose phosphate (PP) pathway consistent with the imposed L-serine overproduction ranged from 0 to 136 mmol gDW^−1^hr^−1^ when constrained by only stoichiometry and from 0 to 62 mmol gDW^−1^hr^−1^ when imposing also kinetic information. This range reduction is due to the limitations of the maximum enzyme activity as well as metabolic concentrations of glucose-6-phosphate (g6p) regulating G6PDH flux. The restrictions implied by kinetics also propagate throughout the stoichiometric part of the network leading to an on average ten percent range contraction for reactions in ***J^stoic^***. For example, the fluxes ranges in 2-oxogluterate dehydrogenase (AKGDH) and succinate dehydrogenase (SUCD) in the TCA cycle decreased 7.4% and 6%, respectively as a direct consequence of the flux range reduction for pyruvate dehydrogenase (PDH) in ***J^kin^*** which supplies acetyl-CoA (accoa) to TCA cycle. As a result of the tighter flux ranges in the overproducing network (and characterization of base strain) many more reactions are identified that must depart from their original ranges (i.e., MUST sets) compared to regular OptForce both in ***MUST^U^*** (38 vs. 3) and ***MUST^L^*** (293 vs. 108) sets. For example, up-regulation of glucose-6-phosphate isomerase (PGI) in upper glycolysis supplies more flux towards 3pg and L-serine production. The flux range for PGI in the overproducing phenotype (−36–100 mmol gDW^−1^hr^−1^) was wide enough to overlap with its reference flux value (35 mmol gDW^−1^hr^−1^) suggesting PGI up-regulation is not necessary for L-serine overproduction. In contrast, using k-OptForce we find that the flux range of PGI in the overproducing phenotype is restricted to 38–98 mmol gDW^−1^hr^−1^ which does not include the reference value of 35 mmol gDW^−1^hr^−1^. This implies that it is impossible to produce L-serine at 90% theoretical yield without directly (or indirectly) increasing the flux through PGI which becomes a member of ***MUST^U^***. We also observe a significant increase in the number of reactions in ***MUST^L^***. This is because the kinetic expressions in ***J^kin^*** fix the branching ratios for fluxes emanating from metabolites in ***I^kin^***. As a result, many reactions in ***J^stoic^*** involving metabolites participating in reactions from ***J^kin^*** appear as down-regulations. For example, k-OptForce identifies that the acetyl-CoA carboxylase (ACCOAC) flux, which branches away from pyr and accoa towards membrane lipid metabolism must decrease (i.e., ***MUST^L^***) as it goes from (9–527) mmol gDW^−1^hr^−1^ in the reference strain to (2.1–2.3) mmol gDW^−1^hr^−1^ in the overproducing strain.

**Figure 7:**
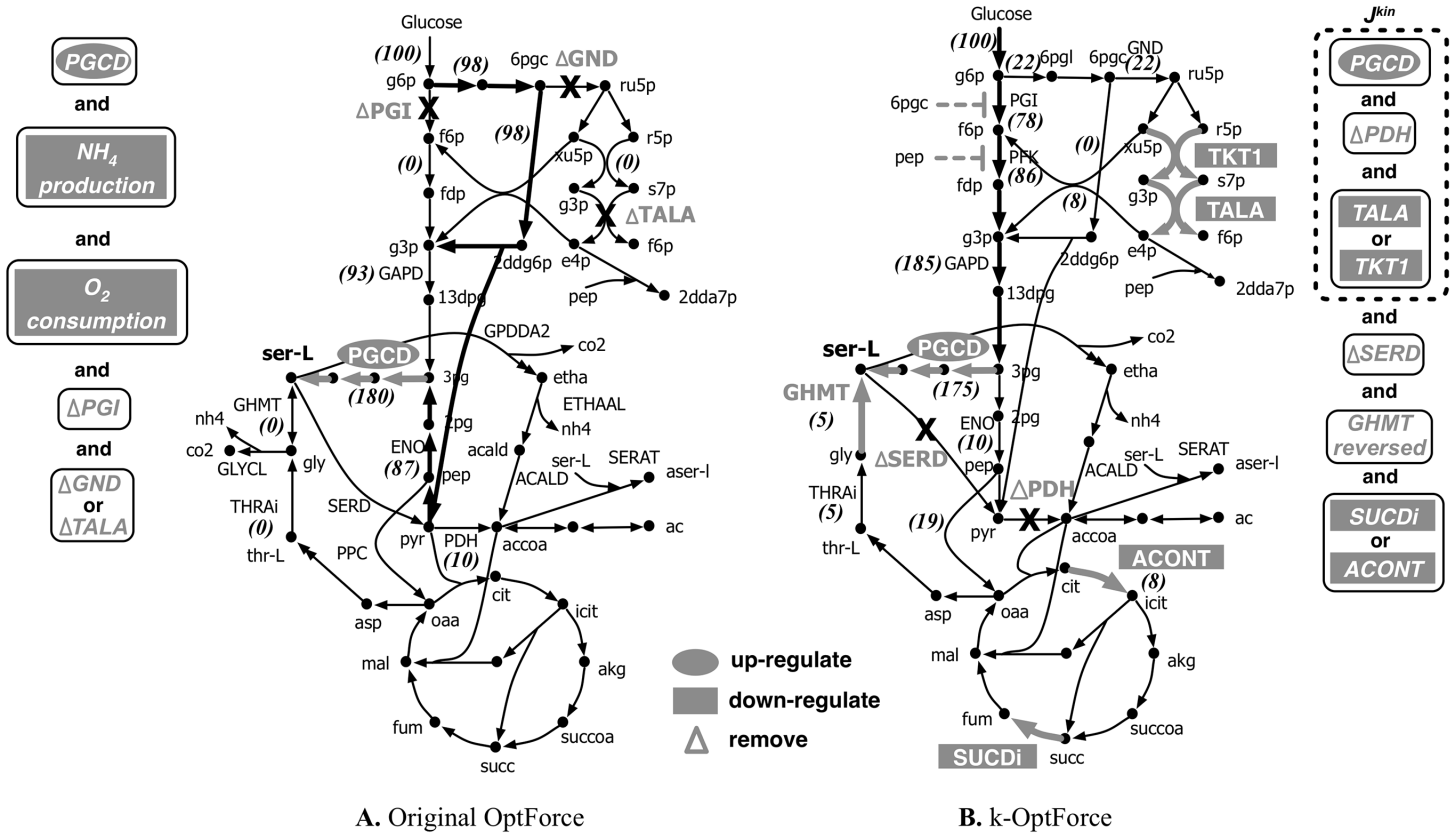
Comparison of intervention strategies predicted by A. regular OptForce and B. k-OptForce for overproduction of L-serine in *E. coli*. The values in brackets indicate the metabolic flux in mmol GDW^−1^hr^−1^ per 100 mmol gDW^−1^ hr^−1^ glucose uptake.

Figure 7 summarizes the FORCE set of reactions as predicted by the original and k-OptForce. As expected, the first intervention suggested by both procedures is an at least 20-fold up-regulation in the activity of one of the three fluxes that directly lead to the synthesis of L-serine (i.e., PGCD, PSERT and PSP). However, the remaining interventions follow completely different strategies. k-OptForce emphasizes the need to remove substrate-level inhibition by making relatively small flux changes on a number of reactions to maintain concentrations within the imposed bounds (i.e., two-fold changes from wild-type measurements). Figure 7b illustrates that it is necessary to up-regulate upper glycolysis and down-regulate lower glycolysis to divert flux towards L-serine. The upper glycolytic pathway is tightly regulated by both product metabolites and nadh [85]. The kinetic expressions in Chassagnole *et al* [76] encode inhibition of PGI and phosphofructokinase (PFK) by 6-Phospho-D-gluconate (6pgc) and phosphoenolpyruvate (pep). The high activity of the PP pathway and lower glycolysis in the wild-type requires elevated intracellular levels of 6pgc (0.8 mM) and pep (2.86 mM) to supply the fluxes through phosphogluconate dehydrogenase (GND) (63 mmol gDW^−1^ hr^−1^) and pyruvate dehydrogenase (PDH) (93 mmol gDW^−1^ hr^−1^) reactions. The high concentrations of 6pgc and pep both prevent the up-regulation of upper glycolysis and the down-regulation of lower glycolysis. To alleviate this, k-OptForce suggests removal of PDH coupled with an at least three-fold down-regulation in the activity of either transaldolase (TALA) or transketolase (TKT1) reactions in PP pathway. Removal of PDH also allows the concentration of pep to be reduced from 2.8 to 1.43 mM thus alleviating its inhibitory effect on PFK. Likewise, down-regulation of the PP pathway activity reduces the flux in GND lowering the concentration of 6pgc (from 0.8 to 0.4 mM). Removal of substrate inhibition on PFK and PGI by pep and 6pgc leads to increased flux towards L-serine. The original OptForce procedure cannot identify such interventions, as substrate inhibition is not captured through stoichiometric modeling.

The inhibitory effect of 6pgc and pep cannot be completely removed due to their prescribed lower limits in concentration (0.4 mM and 1.43 mM respectively). Moreover, the upper limits on concentration of metabolites involved in upper glycolysis put an upper bound on the amount of flux that can be carried by upper glycolysis. Therefore, additional interventions are needed to meet the L-serine target yield by modulating pathways away from glycolysis. k-OptForce suggests the reversal of glycine hydroxymethyltransferase (GHMT) thus converting glycine to L-serine (see Figure 7b). In contrast, the original OptForce predicts that the entire amount of flux required for L-serine can be supplied through the up-regulation of the serine synthase pathway as no inhibitory effect or concentration bound is considered. It is to be noted here that the forward activity of GHMT is essential *in vivo* [86], [87]. If, however, the lower limits on the concentration of 6pgc and pep are reduced to 0.35 mM and 1.3 mM respectively, their inhibitory effect on upper glycolysis is alleviated sufficiently to route all the flux required for L-serine production through the serine synthase pathway. The upper glycolytic flux of PGI increases from 78 to 80 mmol gDW^−1^ hr^−1^ and the PP flux is down-regulated further (from 22 to 20 mmol gDW^−1^ hr^−1^) to provide the extra flux for L-serine (results not shown here). As a result, k-OptForce suggests down-regulation of GHMT by at-least 3 folds from its reference flux instead of its reversal. All other interventions remain un-altered.

The remaining interventions suggested by k-OptForce aim at preventing the drain of metabolic flux from L-serine. Removal of L-serine deaminase (SERD_L) prevents the conversion of L-serine to pyruvate. This is followed by an at least six-fold down-regulation (from 60 to 9 mmol gDW^−1^ hr^−1^) of either citrate synthase (CS) or succinate dehydrogenase (SUCD) reactions to reduce the TCA cycle activity which arrests ATP production in the network. This prevents the conversion of L-serine to acetaldehyde whose activity requires five units of ATP per unit of flux. The original OptForce achieves the same goal by simply down-regulating the transport of oxygen and up-regulating the transport of ammonium into the cell. These interventions were not chosen by k-OptForce as they lead to flux values that are inconsistent with the kinetic expressions in ***J^kin^***.

Consistent with k-OptForce predictions, metabolic engineering studies on *C. glutamicum* have revealed that overexpression of the *serA/B and C* encoding for the three enzymes in the L-serine production pathway have a positive, though small, effect on L-serine production [88], [89]. Removal of *sdaA* encoding for the SERD_L reaction, coupled with up-regulation of the L-serine pathway have been reported to lead to higher L-serine yields [89] consistent with k-OptForce predictions. Other studies have shown that down-regulation of GHMT reaction through the removal of *glyR* regulator further improves L-serine production [90]. In a recent study, overexpression of *pgk* was shown to divert more flux towards L-serine in *C. glutamicum* [91]. This could be viewed as an alternative strategy to the one suggested by k-OptForce involving alleviation of the substrate level inhibition of upper glycolysis through down-regulation of PP and lower glycolytic flux. It must be emphasized that the k-OptForce results depend heavily on the accuracy of the rate expressions of the kinetic model. For example, it has been found in both *E. coli* and *C. glutamicum*, that the activity of PGCD is feedback inhibited by L-serine [81], [92]. Alleviating this feedback regulation significantly improves production of L-serine [91]. However, k-OptForce cannot capture this regulation since the adopted kinetic model does not include this inhibitory effect. Accordingly, k-OptForce predictions must be carefully scrutinized to identify the driving forces for the identified interventions (e.g., substrate inhibition removal, ATP drain, cofactor sequestering, concentration increase, etc.) and the reason for the omission of seemingly straightforward interventions (e.g., concentration bound violation, inadequate *v^max^*, lethal deletion, cofactor imbalance, etc.).

In addition to suggesting intervention strategies consistent with the kinetic constraints in the network, k-OptForce also pinpoints which ones and how original OptForce interventions violate network kinetics. For example, the original OptForce framework suggested the reversal of lower glycolytic reactions that converge to 3pg. This is accomplished by removing PGI and either GND, TKT1 or TALA in PP pathway to reroute the metabolic flux toward 3pg and pyruvate through E-D pathway by using 2-dehydro-3-deoxy-phosphogluconate aldolase (EDA). Reversal of enolase (ENO) and phosphoglycerate mutase (PGM) in lower glycolysis converts pyruvate to 3pg. k-OptForce finds that this redirection is not feasible. Reversible reactions PGM and ENO rely on the relative concentrations of its reactants and products to inform their directionality. Their reversal, to the extent suggested by the original OptForce procedure requires the respective metabolic levels of 2-phosphoglycerate (2pg) and pep to increase to 1.162 and 6.05 mM respectively, beyond the imposed upper limits of (0.856 and 4.726 mM respectively). Therefore, k-OptForce provides both a check on stoichiometry-only derived interventions and more importantly quantifies the impact of flux redirections on metabolite concentrations and required enzyme levels.

### Sensitivity of the k-OptForce results to perturbations in kinetic parameters

Previous reports [93] on the sensitivity analysis of the *E. coli* kinetic model by Chassagnole *et al* [76] showed that simulation results are only sensitive to the values of nine (out of 25) enzyme activities in the model. In light of this analysis, we perturbed the enzyme activities of two sensitive ones (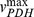 and 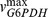) and two rather insensitive (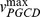 and 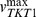) by +/−20% from their reference levels and repeated the k-OptForce calculations. Results showed that apart from 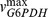 up-perturbation, the remaining interventions (both up-perturbation and down-perturbation) were identical to the original results. Increasing the value of 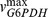, which is one of the most highly sensitive parameters in the model [93], increased the PP flux for the reference phenotype by 11% (from 63 mmol gDW^−1^hr^−1^ to 70 mmol gDW^−1^hr^−1^), as the glycolytic pathway was inhibited by increased 6pgc concentration. As a result, down-regulation of GND was suggested as an additional intervention to reduce the increased PP activity and route more flux from glycolysis towards L-serine. In all other cases metabolite concentrations and fluxes in the kinetic model were minimally affected by perturbations in enzyme activity.

### Production of triacetic acid lactone (TAL) in *S. cerevisiae*

**Figure 8:**
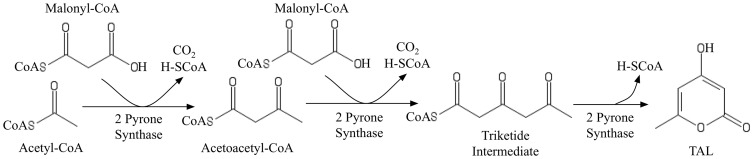
Pathway for TAL production in *S. cerevisiae*.

4-Hydroxy-6-methyl-2-pyrone, commonly known as TAL, is a precursor for the production of phloroglucinol [94], which is an intermediate for many products such as 1,3,5- triamino-2,4,6-trinitrobenzene (TATB) and resorcinol [95]. Synthesis of TAL [96] in both *E. coli* and *S. cerevisiae* has been explored [97] [98]. Because neither *E. coli* nor *S. cerevisiae* can natively synthesize TAL, production routes for TAL rely on the heterologous expression of non-native enzymes such as 2-pyrone synthase (2-PS) (found in *Gerbera hybrida*) [96] or a genetically modified 6-methylsalycilic acid synthase (6-MSAS) [96], [97] and *fasB* [96], [98] with their ketoreductase domains deactivated. These efforts have led to TAL yields in *S. cerevisiae* of only up to 6% of the theoretical maximum (with a titer of 1.8 g/l) in glucose medium. Figure 8 shows the targeted pathway for TAL synthesis in *S. cerevisiae*. We used the *i*AZ900 model [99] of *S. cerevisiae* as the stoichiometric network of metabolism. The kinetic expressions for reactions in central metabolism were imported from the kinetic model of central metabolism of *S. cerevisiae* described by van Eunen *et al* [79]. The model consists of twelve metabolites and twelve reactions, for the glycolytic pathway and the conversion of pyruvate to ethanol. Since the kinetic model did not include drains for amino acids from the central metabolic pathway metabolites (g6p, f6p, g3p, 3pg, pep), we added drains (similar to the method used in Chassagnole *et al* [76]) using MFA information of *S. cerevisiae* central metabolism from Gombert *et al* [100] to ensure biomass production (see Supplementary Material S1).

As in the first example, we allowed for up to two-fold changes in the metabolite concentrations and *v^max^* from their wild-type values. Contrary to the previous example, here the kinetic expressions do not restrict further the flux ranges as the ABC metric (see Methods) for all ***J^kin^*** and ***J^stoic^*** fluxes is zero. As a result, we find no difference in the ***MUST^U^*** (19 reactions) and ***MUST^L^*** (61 reactions) sets for kinetic and original OptForce. This is due to the relatively few fluxes with kinetic expressions and the already fairly tight flux ranges achieved by stoichiometry-alone. For example, the PGI flux varies within the narrow range between 91 and 97 mmol gDW^−1^hr^−1^ for a 90% yield for TAL even when no kinetic expressions are used. This is because the imposed high production target for TAL largely fixes the flow in glycolysis. As a consequence of negligible contraction in flux ranges due to the kinetic constraints, no difference in predicted MUST sets by regular and k-OptForce is observed.

**Figure 9:**
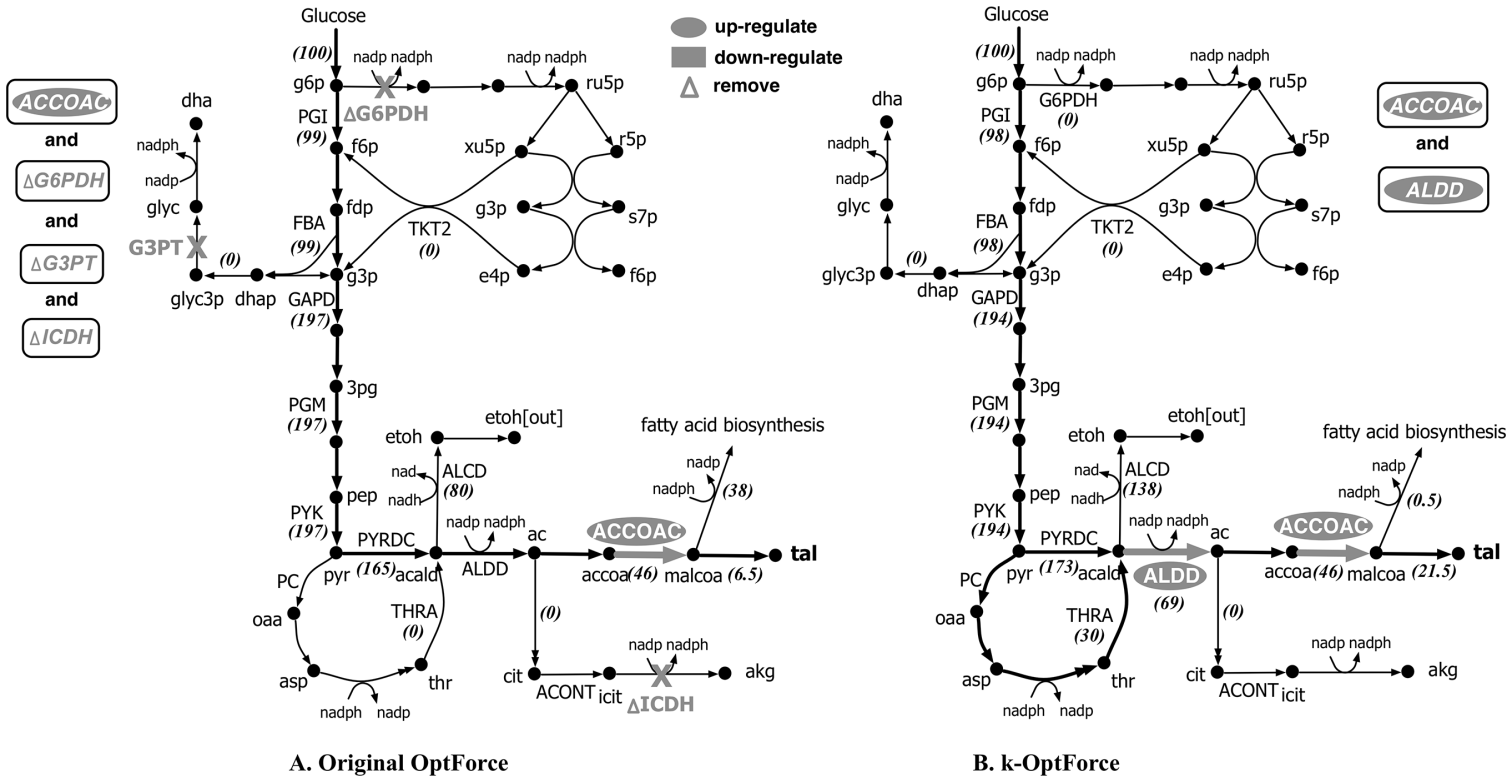
Comparison of intervention strategies predicted by A. regular OptForce and B. k-OptForce for overproduction of TAL in *S. cerevisiae*. The values in brackets indicate the metabolic flux in mmol gDW^−1^ hr^−1^ per 100 mmol gDW^−1^ hr^−1^ glucose uptake.

**Figure 10:**
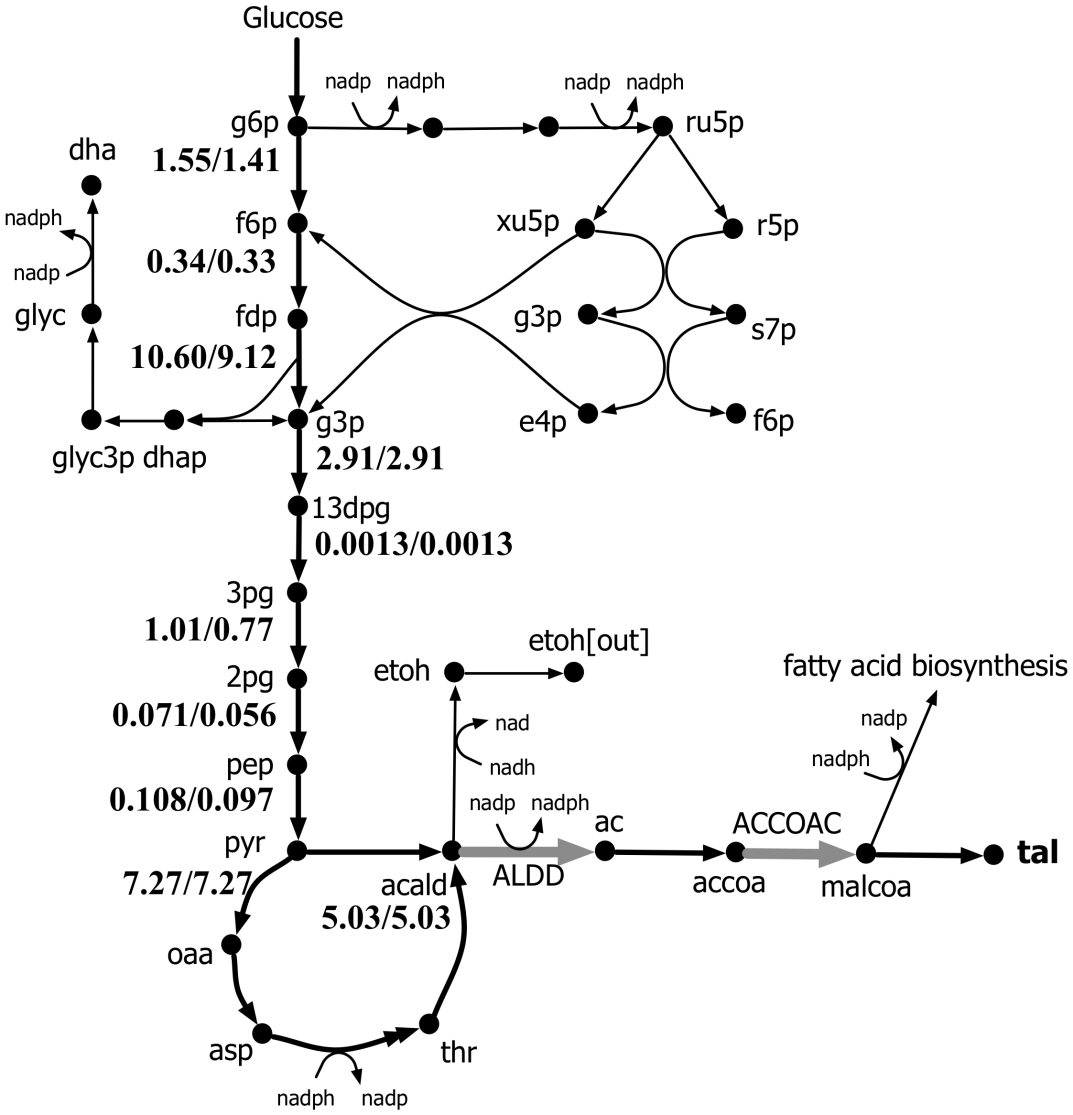
Comparison of the concentrations (in mM) of metabolites in *I^kin^* for the engineered and reference strain of *S. cerevisiae* for overproduction of TAL. The first value indicates the overproducing strain concentration, while the second value refers to the reference strain concentration.

Figure 9 compares the FORCE sets and the respective guaranteed yield of TAL as suggested by original and k-OptForce. In general, both procedures suggest strategies that increase the availability of precursors accoa and malonyl-CoA (malcoa), up-regulating glycolysis, down-regulating PPP, and reducing nadph. However, while the original OptForce suggests that at least four interventions are required to achieve a 35% yield for TAL, k-OptForce suggests that a yield of 90% is achievable by only two interventions. Not surprisingly, both procedures suggest the up-regulation of the ACCOAC (by at least nine-fold of its reference flux) to increase the availability of the direct TAL precursor malonyl-CoA. The glycolytic flux is also up-regulated to divert flux from the PP pathway towards TAL. k-OptForce identifies that the kinetic expressions work in concert with the overproduction goal (given the imposed concentration ranges) without the need for any direct enzymatic interventions for reactions in ***J^kin^***. Figure 10 illustrates the required changes in metabolite concentrations in the overproducing network as predicted by k-OptForce. Elevated concentrations of metabolites in glycolysis lead to an increase the flux towards TAL. For example, the concentration of g6p in the upper glycolysis is increased by 8% (from 1.41 to 1.55 mM) leading to more flux through PGI (from 78 to 96 mmol gDW^−1^ hr^−1^). This eliminates all flux through G6PDH and PP (from 19 to 0 mmol gDW^−1^ hr^−1^) to maintain the steady-state metabolite balance of g6p. Without the benefit of any kinetics, the original OptForce suggests the removal of G6PDH reaction as a requirement for down-regulating the PP pathway. k-OptForce also requires that the concentrations of glyceraldehyde 3-phosphate (g3p) and 3pg be elevated by 6% and 31%, respectively from their reference states to up-regulate the lower glycolysis flux from 168 to 192 mmol gDW^−1^ hr^−1^. This re-direction in glycolysis prevents the loss of metabolic flux towards glycerol synthesis. Instead, the original OptForce procedure suggests the removal of glycerol-3-phosphatase (G3PT) in the glycerol synthesis pathway to serve the same purpose and channel the flux towards lower glycolysis and TAL.

The fatty acid synthase is in direct competition to TAL production. It uses the same precursors as TAL (i.e., accoa and malcoa) to form medium chain fatty acids. Activity of fatty acid synthase requires the cofactor nadph for the reductive steps in the pathway. Not surprisingly, both kinetic and original OptForce identify strategies to lower the availability of nadph. k-OptForce achieves this by suggesting a 20 fold up-regulation (from 3.5 to 69 mmol gDW^−1^ hr^−1^) in the flux of aldehyde dehydrogenase (ALDD) which converts acetaldehyde towards acetate. The major routes of acetaldehyde production in *S. cerevisiae* are either from direct decarboxylation of pyruvate through pyruvate decarboxylase (PYRDC) or through the alternate threonine synthesis pathway followed by the cleavage of threonine by threonine aldolase (THRD) to acetaldehyde and glycine (see Figure 9B). The threonine synthesis pathway is favored in TAL overproduction as it consumes one unit of nadph for every unit of flux. We note that the fluxes in PYRDC and alcohol dehydrogenase (ALCD) are fixed by the kinetic constraints in ***J^kin^***. Therefore, up-regulation of ALDD causes most of pyruvate to be routed through the threonine production pathway (to maintain the steady-state conservation of acetaldehyde) resulting in a decrease in nadph levels. The original OptForce does not arrive at this intervention as the kinetic control on the fluxes of PYRDC and ALCD is not captured. It instead suggests the removal of cytosolic isocitrate dehydrogenase (ICDHy) to reduce the nadph production, and thus arrests fatty acid synthesis.

Unlike the previous example where k-OptForce required more interventions for the same overproduction target than the original OptForce, here the reverse trend is observed. The predicted yield for TAL by the original OptForce is only 35% of its theoretical maximum after four interventions whereas k-OptForce reaches 90% of theoretical maximum with only two manipulations. This is because the incorporation of kinetic information pushes metabolic flux in the direction that is needed for overproduction and away from the “worst-case” behavior.

### Effect of metabolite concentration ranges on identified interventions

The steady-state balances of metabolites in a kinetic model [76], [79] (with as many metabolites as rate equations) form a square-system of equations with zero degrees of freedom. Assuming that there are no multiple steady-states due to the nonlinearity of the kinetic expressions, steady-state fluxes or concentrations cannot change unless accompanied by alterations in kinetic parameters (e.g. enzyme activities *v^max^*). However, when a kinetic model is integrated with a stoichiometric genome-scale model, reactions in ***J^stoic^*** that involve metabolites present in ***I^kin^*** add in effect additional degrees of freedom to the square-system of equations thus decoupling metabolite concentrations from enzyme activities. As a result metabolite concentrations can change in such a way that fluxes are altered without requiring any enzymatic interventions as observed in the TAL overproduction example. In the absence of kinetic expressions for all reactions associated with the metabolites in ***I^kin^*** a number of degrees of freedom remain for the metabolite concentrations. To avoid drastic concentration changes in response to the overproduction goal we explored penalizing deviations of metabolite concentrations from their reference steady-state values using a weight penalty factor *ε*. This posture in essence imposes a homeostasis term in the optimization objective function. The outer objective function of the bilevel formulation for identifying FORCE sets is thus modified as follows:
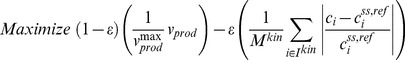
The first term in the objective function is the flux of the desired chemical 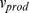, scaled by its theoretical maximum flux in the network 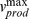. The second term is the average fractional departure of the metabolites in ***I^kin^*** (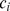) from their reference values (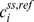). M^kin^ represents the total number of metabolites in ***I^kin^***. When using the two-step procedure for identifying MUST sets (see Methods and Supplementary Material S1), the objective function for the first step is modified as follows:
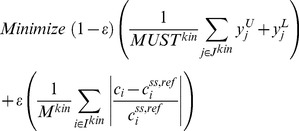
In this objective function, the first term is the sum of interventions in ***J^kin^***, scaled by the total number of reactions in ***J^kin^*** identified in MUST sets (***MUST^kin^***). No other changes are made in the formulation of the algorithms. We tested this modified formulation on TAL production in *S. cerevisiae*.

**Figure 11:**
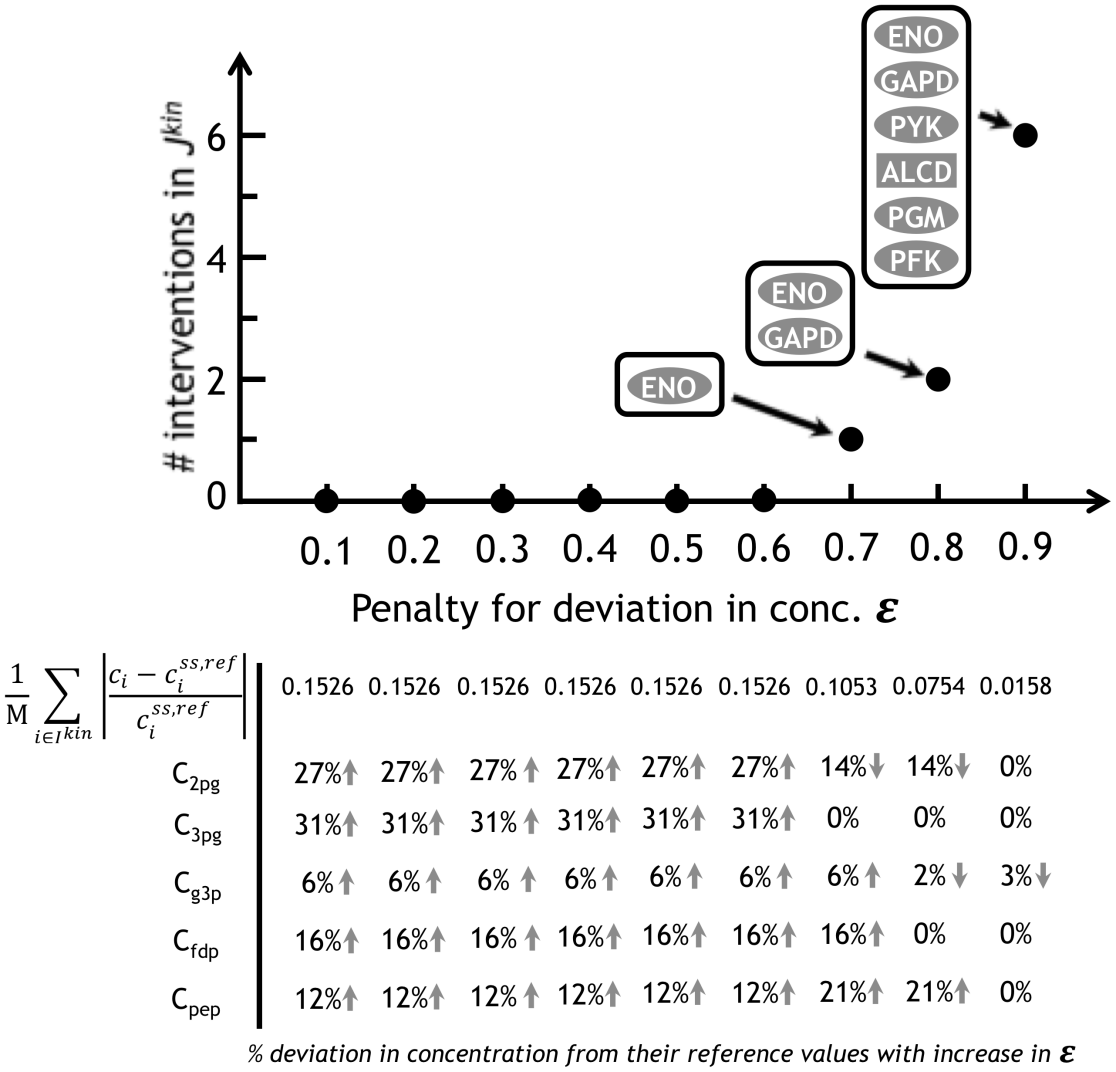
Predicted interventions in *J^kin^* for TAL overproduction in *S. cerevisiae* as a function of penalty factor (ε). The graph above shows the variation in the minimum number of interventions required in ***J^kin^*** to overproduce TAL with increase in ε. The corresponding table below shows of the average deviation (row 1) and the individual deviation in concentration of metabolites in ***I^kin^*** for different values of ε.

Figure 11 describes the effect of penalizing concentration departures on enzymatic interventions in ***J^kin^*** for the overproduction of TAL in *S. cerevisiae*. We varied ε from 0.1 (low penalty) to 0.9 (very high penalty) on the identified interventions. Up to a ε value of 0.6, the penalty is not high enough to require direct interventions instead of concentration changes. For a ε value of 0.7 k-OptForce identified up-regulation of enolase (ENO) while maintaining the average deviation in concentration to 0.1053. Note that without the use of the penalty term the concentrations for 3pg and 2pg have to be elevated by 31% and 27% respectively, from their reference levels to redirect more flux through the lower glycolytic reactions of glyceraldehyde-3-phosphate dehydrogenase (PGM) and ENO. For ε = 0.8 the metabolite concentrations remain even closer to their reference levels (average deviation is 0.0754) thereby requiring the up-regulation of glyceraldehyde-3-phosphate dehydrogenase (GAPD) in addition to ENO. For ε equal to or greater than 0.9 at least 6 additional enzymatic interventions in ***J^kin^*** are necessary to increase glycolytic flux while concentrations remain very close to their reference values. By increasing the value of the penalty ε enables drawing trade-offs between allowable concentration changes and minimality of needed interventions for an overproduction goal while also providing a prioritization strategy for implementing engineering interventions.

### Expression of a heterologous enzyme in *S. cerevisiae* to improve TAL yield

The conversion from cytosolic pyruvate to acetyl-CoA (precursor for TAL) in yeast follows a long and tightly regulated path involving the intermediate production of acetaldehyde and acetate [101]. We sought to computationally explore the use of a direct route from pyruvate to acetyl-CoA by adding a heterologous cytosolic PDH from *E. coli* in *S. cerevisiae* that directly converts pyruvate to acetyl-CoA. Note that *S. cerevisiae* has a pyruvate dehydrogenase activity in mitochondria but not in cytosol. The PDH complex in *E. coli* uses nad as the cofactor, however, an nadp-dependent PDH enzyme (constructed by site-directed mutagenesis in the 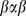 fold of the nad-binding domain of dihydrolipoamide dehydrogenase) has also been expressed in *E. coli* with identical kinetic properties [102]. The maximum theoretical yield of TAL using the nadp-dependent PDH enzyme increased by 40%. By bypassing the multi-step conversion of pyruvate to acetyl-CoA, two ATP equivalents of energy are conserved. No such maximum yield improvements are found for the nad-dependent PDH due to nad imbalance in the cytosol.

**Figure 12:**
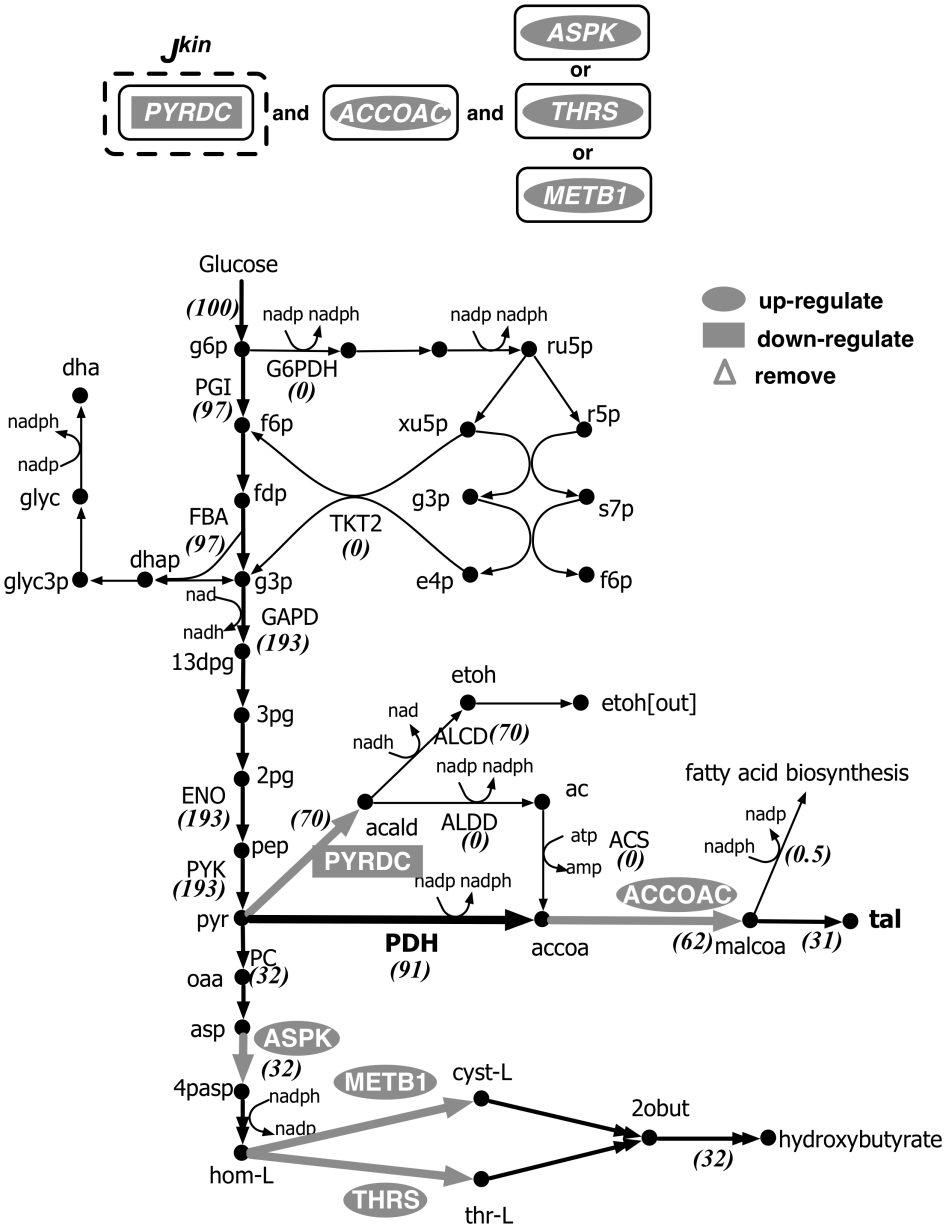
Metabolic interventions predicted by k-OptForce for overproduction of TAL in *S. cerevisiae* on heterologous expression of nadph-dependent PDH complex from *E. coli*. The values in brackets indicate the metabolic flux in mmol gDW^−1^hr^−1^ per 100 mmol gDW^−1^ hr^−1^ glucose uptake.

The kinetic expression for PDH was extracted from the kinetic model of *E. coli* proposed in Chassagnole *et al.* [76]. The interventions predicted by k-OptForce for maximizing TAL production are shown in Figure 12. Upon addition of the heterologous PDH, the entire amount of flux towards TAL production is routed through PDH. This eliminates the ACS activity that previously drained ATP. Pyruvate decarboxylase (PYRDC) is now down-regulated, but its activity is not reduced to zero. The entire flux of PYRDC goes towards ethanol production to regenerate NAD and maintain the cofactor balance in cytosol [101]. Instead of up-regulating ALDD, k-OptForce identifies an alternative intervention to lower nadph availability by up-regulating either aspartate kinase (ASPK), threonine synthase (THRS) or cystathionine synthase (METB1) in the hydroxybutyrate production pathway.

## Discussion

k-OptForce integrates kinetic relations (whenever available) with stoichiometry based models to identify genetic perturbations that are consistent with enzyme expressions and metabolite concentrations. The resulting optimization problems pose significant computational challenges due to the bilevel nature of the formulation and the nonconvex terms in the objective function and constraints. We introduced tractable solution workflows for recasting the problems as equivalent single-level mixed-integer nonlinear optimization problems (MINLP) solved using the global optimization solver BARON to optimality. A hierarchical decomposition approach is also introduced for first identifying interventions within the kinetic part of the model followed by the interventions in the stoichiometry-only part of the model. As with other computational algorithms that make use of kinetics the results can be dependent upon the kinetic model structure and parameterization.

Computational results show that the introduction of kinetic expressions in strain design can significantly affect the identified interventions in sometimes non-intuitive ways. In some cases additional modifications are needed to substitute interventions that cause enzyme saturation or concentration bound violations. The mechanism of action of these modifications is often subtle by alleviating substrate inhibition or draining away cofactors from competing pathways. In other cases, kinetic expressions shape flux distributions so as to favor the overproduction of the desired product requiring fewer direct interventions. Uncertainties in both the accuracy of the kinetic models and allowable concentration ranges imply that predicted interventions need to be carefully scrutinized to pinpoint the reasons for their inclusion. An important finding in this study was that concentration ranges have a very significant effect on the identified interventions. By penalizing departures of concentrations from the reference strain values substantial re-arrangements in the predicted interventions are observed. Each one of these changes can be analyzed and the underlying reason for its inclusion can be identified.

A key contribution of kinetic descriptions is that they can attribute performance bottlenecks to specific concentration bounds and/or enzymatic parameter ranges bottlenecks revealing avenues for model improvement and strain optimization. The case study for L-serine overproduction in *E. coli* provides an example of how k-OptForce can be used to both identify interventions and trace the reason(s) for the exclusion of others. k-OptForce revealed that inhibition of upper glycolysis by pep and 6pgc must be alleviated to route more flux towards L-serine. This is achieved through removal of PDH and down-regulation of TALA or TKT1 respectively. Flux analysis on single gene mutant strains of *E. coli* show that deletion of either *tala* or *tktA* increases the flux through PGI [103] corroborating k-OptForce predictions. However, MFA data for the *lpdA* mutant encoding the PDH enzyme in *E. coli* [104] showed that the upper glycolysis is down-regulated and that the flux through PP pathway is up-regulated, contrary to k-OptForce predictions. A possible reason for this discrepancy could be due to insufficiencies in the kinetic expressions used to describe the reactions in ***J^kin^***. Alternatively, since *lpdA* also encodes for the activity of ICDHy and the glycine cleavage system (GLYCL), its removal could be have a combined effect on down-regulating the flux in upper glycolysis, which is not captured by the kinetic model. k-OptForce, however, correctly predicts that PDH removal down-regulates lower glycolysis which is observed in the *lpdA* mutant strain [104]. Down-regulation of lower glycolysis is necessary to prevent the flux towards L-serine from draining away towards pyruvate. k-OptForce also sets an upper limit on the activity of the L-serine synthase pathway that the original OptForce procedure failed to pinpoint. In addition, k-OptForce prevents rearrangement of fluxes that would violate kinetic constraints and metabolite concentration limits. The original OptForce suggested reversal of lower glycolysis by rerouting metabolic flux through ED pathway. However, such re-distribution results in the upper and lower glycolysis to operate in opposite directions which cannot be achieved as the same regulator, (i.e., *cra*), determines the directionality of both upper (i.e., PFK) and lower glycolysis (i.e., PYK) [105], [106] and represses the ED pathway upon reversal of glycolysis.

k-OptForce may require fewer direct interventions if the kinetic expressions shape fluxes so as to favor the desired overproduction product as observed for the production of TAL where up-regulation of ALDD was suggested to redirect flux from pyruvate to acetyl-CoA. This is consistent with an experimental study for isoprenoid overproduction in *S. cerevisiae* [107] which demonstrated that overexpression of *ald6* (which encodes for the ALDD enzyme) increases flux towards acetyl-CoA. However, a fraction of the flux from pyruvate to acetaldehyde was routed through threonine degradation without the requirement of any additional interventions. This direct intervention-free flux redistribution may be an artifact of the kinetic model and may require direct manipulations to engineer. Metabolome studies on single-gene mutant analysis in *E. coli* [103] revealed that, on average, internal metabolite concentrations were minimally altered from their reference concentrations as a result of the genetic perturbations. Changes in metabolic fluxes were largely the result of changes in enzyme activities. In response to this we postulated the use of a penalty term for violating homeostasis of metabolite levels. Alternatively, one could employ the method described in Smallbone *et al* [73] to formulate approximate lin-log expressions for all reactions associated with metabolites in ***I^kin^*** that do not have a kinetic expression (i.e. not part of ***J^kin^***). This would restore the square-system of equations in ***J^kin^*** and recouple all metabolite concentrations with enzyme activities.

The k-OptForce procedure is versatile enough to incorporate additional omics information, whenever available, to further improve prediction fidelity. For example, MFA data for reactions can be included as additional constraints to further tighten flux ranges. k-OptForce can also capture other types of metabolic regulation and select from a wider palette of direct interventions (e.g., enzymatic changes and transcriptional control) such as the dynamic hybrid model of *E. coli* metabolism by Lee *et al* [108] that integrates signaling and transcriptional regulation with FBA. Temporal consideration can also be addressed be deploying k-OptForce within the dFBA framework [65] to explore the variation of metabolic interventions as a function of time alluding to RNAi type of interventions. We expect that k-OptForce predictions will help improve the breadth and accuracy of kinetic modeling descriptions by providing the quantitative means to assess model accuracy ultimately leading to improved fidelity of metabolic descriptions.

## Supporting Information

Text S1 Optimization formulation and solution procedure of k-OptForce, kinetic models of central metabolism for *E. coli* and *S. cerevisiae*, supplementary figures for steady-state flux distribution and metabolite concentrations for the kinetic models, and flux ranges for the reference and overproducing phenotypes for L-serine and TAL production.
(PDF)
