# Supplementary material for: k-OptForce: Integrating Kinetics with Flux Balance Analysis for Strain Design
Source: PLoS Comput Biol. 2014 Feb 20;10(2):e1003487. doi: 10.1371/journal.pcbi.1003487 (PMC3930495; doi:10.1371/journal.pcbi.1003487)
Supplement: Text S1 — Optimization formulation and solution procedure of k-OptForce, kinetic models of central metabolism for E. coli and S. cerevisiae, supplementary figures for steady-state flux distribution and metabolite concentrations for the kinetic models, and flux ranges for the reference and overproducing phenotypes for L-serine and TAL production. (PDF) [file pcbi.1003487.s001.pdf]

k-OptForce: Integrating kinetics with flux balance analysis for strain design

Authors: Anupam Chowdhury, Ali R. Zomorrodi, and Costas D. Maranas

Department of Chemical Engineering, Pennsylvania State University, University Park, PA

Mathematical description of k-OptForce

The k-OptForceprotocol for strain engineering requires the definition of the following sets, parameters and variables:

Sets:

$\boldsymbol{I}=\{i|i=1,2,\ldots,M\}$ = Set of metabolites in the stoichiometric model

$\boldsymbol{J} =\{j\ |j =1,2,\ldots,N\}$ = Set of reactions in the stoichiometric model

$\boldsymbol{J^{kin}} = \{j\ |j =1,2,\ldots,N^{kin}\}$ = Subset of reactions which are part of the kinetic model

$\boldsymbol{J^{stoic}} = \{j\ |j =1,2,\ldots,N^{stoic}\}$ = Subset of reactions described only by stoichiometry

$\boldsymbol{I^{upt}} = \{i|i=1,2,\ldots,M^{upt}\}$ = Set of metabolites that are taken up by the organism

$\boldsymbol{I^{kin}} = \{j\ |i=1,2,\ldots,M^{kin}\}$ = Subset of metabolites involved in reactions in $\boldsymbol{J^{kin}}$

$\boldsymbol{P} = \{p|p =1,2,\ldots,P\}$ = Set of parameters for kinetic expressions for reactions in $\boldsymbol{J^{kin}}$

$\boldsymbol{MUST^{U}}$ = Set of reactions that must be up-regulated in reference strain to ensure target product overproduction

$\boldsymbol{MUST^{L}}$ = Set of reactions that must be down-regulated in reference strain to ensure target product overproduction

$\boldsymbol{MUST^{X}}$ = Set of reactions that must be removed from reference strain to ensure target product overproduction

$$\boldsymbol{MUST^{stoic}} = \boldsymbol{J^{stoic}} \cap \{\boldsymbol{MUST^{U}} \cup \boldsymbol{MUST^{L}} \cup \boldsymbol{MUST^{X}}\}$$

$\boldsymbol{B^{U}}$ = Set of reactions with finite upper bounds (i.e., reactions for uptake of nutrients)

$\boldsymbol{B^L}$ = Set of reactions with finitelower bounds (i.e., irreversible reactions and flux towards biomass)

Parameters:

S_{ij} = Stoichiometric coefficient of metabolite $i \in \boldsymbol{I}$ in reaction $j \in \boldsymbol{J}$

UB_j = Upper bound for the flux of reaction $j \in \boldsymbol{J}$

LB_j = Lower bound for the flux of reaction $j \in \boldsymbol{J}$

v_j^{ss} = Steady-state flux for reactions $j \in \boldsymbol{J^{kin}}$ in the reference strain obtained by solving the system of ODEs

$v_j^{U,kin}$ = Upper bound of flux for reaction $j \in \boldsymbol{J^{kin}}$in the overproducing strain

$v_j^{L,kin}$ = Lower bound of flux for reaction $j \in \boldsymbol{J^{kin}}$in the overproducing strain

$v_j^{max,ref}$ = The maximum enzymatic reaction rate of reaction j belonging to $\boldsymbol{J^{kin}}$ in the reference strain

u_i^{upt} = Value of uptake flux of metabolite $i \in \boldsymbol{I^{upt}}\left(u_i \in \{v_j\}\right)$

c_i^{max} = Lower bound on concentration for metabolite $i \in \boldsymbol{I^{kin}}$ constructed by allowing for $+50\%$ deviation in their steady-state concentrations (from solving the ODEs)

c_i^{min} = Lower bound on concentration for metabolite $i \in \boldsymbol{I^{kin}}$ constructed by allowing for -50% deviation in their steady-state concentrations (from solving the ODEs)

z = The maximum allowable fold change on $v_j^{max,ref}$ for $j \in \boldsymbol{J^{kin}}$

k_j^p = Value for kinetic parameter $p \in \boldsymbol{P}$ associated with reaction $j \in \boldsymbol{J^{kin}}$

$v_{biomass}^{max,ref}$ = Maximum flux of biomass production in the reference strain consistent with steady-state fluxes $v_j^{ss}$for $j \in \boldsymbol{J^{kin}}$

$v_{biomass}^{min}$ = Minimum required flux of biomass production in the engineered strain

v_{prod}^{target} = Minimum flux of target chemical production in the engineered strain

$v\left(v_j^{max}, c_i, k_j^p\right)$ = Kinetic expression describing the flux of reaction $j \in \boldsymbol{J^{kin}}$, as a function of v_j^{max},c_i and k_j^p

Variables:

v_j = Flux of reaction j,for all $j \in \boldsymbol{J}$

$v_{biomass}$ = Flux of reaction producing biomass

v_{prod} = Flux of reaction producing the target chemical

u_i = Uptake flux of metabolite $i \in \boldsymbol{I^{upt}} (u_i \in \{v_j\})$

v_j^{max} = The maximum enzymatic reaction rate of reaction $j \in \boldsymbol{J^{kin}}$in the engineered strain

c_i = Concentration for metabolite $i \in \boldsymbol{I^{kin}}$

r_j = Flux of reaction j,for all $j \in \boldsymbol{J^{kin}}$

δ_j^U = Dual variable associated with constraint involving $y_j^{U,stoic}$, for all $j \in \left\{\boldsymbol{J^{stoic}} \cap \boldsymbol{MUST^U}\right\}$

δ_j^L = Dual variable associated with constraint involving $y_j^{L,stoic}$, for all $j \in \left\{\boldsymbol{J^{stoic}} \cap \boldsymbol{MUST^L}\right\}$

η_j^U, η_j^L= Dual variables associated with constraints involving $y_j^{X\ stoic}$, for all $j \in \{\boldsymbol{J^{stoic}} \cap \boldsymbol{MUST^X}\}$

γ_j^U = Dual variable associated with constraint on lower bound on v_j, for all $j \in \boldsymbol{J}$

γ_j^L = Dual variable associated with constraint on upper bound on v_j, for all $j \in \boldsymbol{J}$

g_j^U = Slack on "less than" primal constraints,for all $j \in \left\{\boldsymbol{B^U} \cup \left\{\boldsymbol{J^{stoic}} \cap \{\boldsymbol{MUST^L} \cup \boldsymbol{MUST^X}\}\right\}\right\}$

g_j^L=Slack on "greater than" primal constraints,for all $j \in \left\{\boldsymbol{B^L} \cup \left\{\boldsymbol{J^{stoic}} \cap \{\boldsymbol{MUST^U} \cup \boldsymbol{MUST^X}\}\right\}\right\}$

α_j^U = Slack on dual variables,for all $j \in \left\{\boldsymbol{B^U} \cup \left\{\boldsymbol{J^{stoic}} \cap \{\boldsymbol{MUST^L} \cup \boldsymbol{MUST^X}\}\right\}\right\}$

α_j^L = Slack on dual variables,for all $j \in \left\{\boldsymbol{B^L} \cup \left\{\boldsymbol{J^{stoic}} \cap \{\boldsymbol{MUST^U} \cup \boldsymbol{MUST^X}\}\right\}\right\}$

Binary variables:

$y_j^{U,kin}$ = Binary variable associated with fluxv_j , for all $j \in \{\boldsymbol{J^{kin}} \cap \boldsymbol{MUST^U}\}$

$y_j^{L,kin}$ = Binary variable associated with fluxv_j , for all $j \in \{\boldsymbol{J^{kin}} \cap \boldsymbol{MUST^L}\}$

$y_j^{U,stoic}$ = Binary variable associated with each fluxv_j , for all $j \in \{\boldsymbol{J^{stoic}} \cap \boldsymbol{MUST^U}\}$

$y_j^{L,stoic}$ = Binary variable associated with each fluxv_j , for all $j \in \{\boldsymbol{J^{stoic}} \cap \boldsymbol{MUST^L}\}$

$y_j^{X,stoic}$ = Binary variable associated with each fluxv_j , for all $j \in \{\boldsymbol{J^{stoic}} \cap \boldsymbol{MUST^X}\}$

The stepwise procedure of the sequence of optimization formulation of k-OptForce is described in the following sections.

Characterization of the phenotype of the reference strain

The optimization formulation for characterizing the feasible space for the wild-type network is described as follows:

$$\left\{ \begin{array}{llll} max/min & v_j & & \\ s.t. & \sum_{j \in J}^{N} S_{ij} v_j = 0, & \forall i \in \boldsymbol{I} & (1) \\ & u_i = u_i^{upt}, & \forall i \in \boldsymbol{I^{upt}} & (2) \\ & LB_j \le v_j \le UB_j, & \forall j \in \boldsymbol{J} & (3) \\ & v_j = v_j^{ss}, & \forall j \in \boldsymbol{J^{kin}} & (4) \\ & v_{biomass} = v_{biomass}^{max,ref} & & (5) \end{array} \right\} \forall j \in \boldsymbol{J}$$

Here,the flux of each reaction in the network is iteratively maximized and minimized subject to limitations of stoichiometry (1), metabolite uptakes (2) and the limits on fluxes (3).In addition, the fluxes for reactions belonging to $\boldsymbol{J^{kin}}$ are set at their steady-state valuesv_j^{ss} (4), obtained from solving the system of ODEs of the kinetic model. The biomass flux$v_{biomass}$ is fixed at the maximum attainable value$v_{biomass}^{max\,,ref}$ (5), consistent with the steady-state flux distributionv_j^{ss} for$j \in \boldsymbol{J^{kin}}$. The iterative set of Linear Programs (LP) are solved using the CPLEX solver accessed through GAMS to global optimality.

Characterization of the phenotype of the overproducing strain

The optimization formulation for defining the feasible space of the engineered strain consistent with the overproduction of the target product is similarly defined as follows:

$$
\left\{
\begin{array}{llrr}
\text{max/min} & v_j & & \\
\text{s.t.} & \sum_{j \in J}^{N} S_{ij} v_j = 0, & \forall i \in \boldsymbol{I} & (1) \\
 & u_i = u_i^{upt}, & \forall i \in \boldsymbol{I^{upt}} & (2) \\
 & v_{biomass}^{min} \leq v_{biomass}, v_{pdt}^{target} \leq v_{prod}, & & (3) \\
 & LB_j \leq v_j \leq UB_j, & \forall j \in \boldsymbol{J} & (4) \\
 & v_j = v\left(v_j^{max}, c_i, k_j^p\right), & \forall i \in \boldsymbol{I^{kin}}, j \in \boldsymbol{J^{kin}}, p \in \boldsymbol{P} & (5) \\
 & c_i^{min} \leq c_i \leq c_i^{max}, & \forall i \in \boldsymbol{I^{kin}} & (6) \\
 & 0 \leq v_j^{max} \leq z.\, v_j^{max,ref} & \forall j \in \boldsymbol{J^{kin}} & (7)
\end{array}
\right\} \forall j \in \boldsymbol{J}
$$

In this formulation, in addition to the constraints of stoichiometry (1), metabolite uptakes (2) and flux ranges (4), Constraint 5defines the kinetic relations for $j \in \boldsymbol{J^{kin}}$ as a function of their maximum activity v_j^{max}, metabolite concentrationsc_i, and other kinetic parameters k_j^p. Since the internal cellular environment of the engineered strain will deviate significantly from their wild-type conditions, the metabolite concentrations and maximum enzymatic activities can no longer be set at their steady-state values for the reference strain. Constraint 6 allows the metabolite concentrations c_i to vary within a range from their steady-state concentrations$\left(c_i^{min} \text{ and } c_i^{max}\right)$ obtained from solving the system of ODEs. The maximum enzyme activity v_j^{max} is allowed to range from zero to a pre-specified factor z times its wild-type value $v_j^{max,ref}$ through (7).

Identification of FORCE sets

The set of minimum manipulations required to guarantee a pre-specified yield of the target chemical (i.e. the FORCE sets) could be obtained from either of the two optimization formulations, which are described as follows:

1. *Single-step formulation:*

This formulation solves a single optimization problem to identifyminimum set of direct engineering interventions, both from $\boldsymbol{J^{kin}}$ and $\boldsymbol{J^{stoic}}$, leading to an increase, decrease or elimination of a reaction flux. Manipulation of fluxes in $\boldsymbol{J^{kin}}$ and $\boldsymbol{J^{stoic}}$ are controlled by separate binary variables.Binary variables $y_j^{U,kin}$ and $y_j^{L,kin}$ propagate the up-regulation and down-regulation of fluxes $\boldsymbol{J^{kin}}$ respectively, while $y_j^{U,stoic}$, $y_j^{L,stoic}$ and $y_j^{X,stoic}$ control the up-regulation, down-regulation and removal of their respective fluxes in $\boldsymbol{J^{stoic}}$.

A max-min bilevel optimization problem is used to identify alternative sets of κ (pre-specified) engineering interventions that maximize the minimum product formation (worst-case scenario) in the network. This formulation can be expressed in the following:

$$\max \quad v_{prod}$$

$$s.t. \quad \sum_{j \in J^{kin}} y_j^{U,kin} + y_j^{L,kin} + \sum_{j \in J^{stoic}} y_j^{U,stoic} + y_j^{L,stoic} + y_j^{X,stoic} = \kappa, \qquad (1)$$

$$y_j^{U,kin} + y_j^{L,kin} \leq 1, \qquad \forall j \in \boldsymbol{J^{kin}} \qquad (2)$$

$$y_j^{U,stoic} + y_j^{L,stoic} + y_j^{X,stoic} \leq 1, \qquad \forall j \in \boldsymbol{J^{stoic}} \qquad (3)$$

$$r_j = v\left(v_j^{max}, c_i, k_j^p\right), \qquad \forall i \in \boldsymbol{I^{kin}}, j \in \boldsymbol{J^{kin}}, p \in \boldsymbol{P} \qquad (4)$$

$$c_i^{min} \leq c_i \leq c_i^{max}, \qquad \forall i \in \boldsymbol{I^{kin}} \qquad (5)$$

$$v_j^{max,ref}\left(1 - y_j^{L,kin}\right) \leq v_j^{max} \leq v_j^{max,ref}, \qquad \forall j \in \{\boldsymbol{J^{kin}} \cap \boldsymbol{MUST^L}\} \qquad (6)$$

$$v_j^{max,ref} \leq v_j^{max} \leq v_j^{max,ref}\left(1 + (z-1)y_j^{U,kin}\right), \qquad \forall j \in \{\boldsymbol{J^{kin}} \cap \boldsymbol{MUST^U}\} \qquad (7)$$

$$v_j^{max} = v_j^{max,ref}, \qquad \forall j \in \{\boldsymbol{J^{kin}} \cap NOT(\boldsymbol{MUST^U} \cup \boldsymbol{MUST^L})\} \qquad (8)$$

$$\min \quad v_{prod}$$

$$\sum_{j \in J}^{N} S_{ij} v_j = 0, \qquad \forall i \in \boldsymbol{I} \qquad (9)$$

$$u_i = u_i^{upt}, \qquad \forall i \in \boldsymbol{I^{upt}} \qquad (10)$$

$$v_{biomass} \geq v_{biomass}^{min}, \qquad (11)$$

$$LB_j \leq v_j \leq UB_j, \qquad \forall j \in \boldsymbol{J} \qquad (12)$$

$$v_j \geq v_j^{OS,L} + LB_j\left(1 - y_j^{U,stoic}\right), \qquad \forall j \in \{\boldsymbol{J^{stoic}} \cap \boldsymbol{MUST^U}\} \qquad (13)$$

$$v_j \leq v_j^{OS,U} + UB_j\left(1 - y_j^{L,stoic}\right), \qquad \forall j \in \{\boldsymbol{J^{stoic}} \cap \boldsymbol{MUST^L}\} \qquad (14)$$

$$LB_j\left(1 - y_j^{X,stoic}\right) \leq v_j \leq UB_j\left(1 - y_j^{X,stoic}\right), \quad \forall j \in \{\boldsymbol{J^{stoic}} \cap \boldsymbol{MUST^X}\} \; (15)$$

$$v_j = r_j, \qquad \forall j \in \boldsymbol{J^{kin}} \qquad (16)$$

$$y_j^{U,kin}, y_j^{L,kin}, y_j^{U,stoic}, y_j^{L,stoic}, y_j^{X,stoic} \in \{0,1\}$$

The inner optimization problem simulates the "worst-case" scenario for the production of the targeted product while the outer part aims to maximize the overproduction target for a certain number of engineered modifications κ(1). Constraints 2 and 3 ensure that a single reaction cannot be targeted for more than one manipulation.The outer problem fixes the flux of the reactions in $\boldsymbol{J^{kin}}$ using (4).The enzymatic manipulations in $\boldsymbol{J^{kin}}$ are identified by Constraints 6-7. v_j^{max} of reactions belonging to both $\boldsymbol{J^{kin}}$ and $\boldsymbol{MUST^{L}}$ sets are constrained by (6), such that for $y_j^{L,kin}$ values of 0, v_j^{max} equals $v_j^{max,ref}$, otherwisev_j^{max} is free to explore any value between zero and $v_j^{max,ref}$, indicating down-regulation of its activity. Likewise, Constraint 7 up-regulates an enzyme belonging to $\boldsymbol{MUST^{U}}$ sets such that it can range from $v_j^{max,ref}$ to $z.v_j^{max,ref}$ overexpression if its corresponding binary $y_j^{U,kin}$ is one, and equals $v_j^{max,ref}$ otherwise.v_j^{max} of reactions in $\boldsymbol{J^{kin}}$ outside the MUST sets are kept at their reference values$v_j^{max,ref}$ using (8). Constraints 12-14 in the inner problem propagate the effect of engineering modifications encoded within $y_j^{U,stoic}$, $y_j^{L,stoic}$ and $y_j^{X,stoic}$ to the corresponding reaction fluxes in $\boldsymbol{J^{stoic}}$. For example, if a reaction is selected for up-regulation then the flux for this reaction is set greater than the minimum value allowed in the overproducing strain ($v_j^{OS,L}$). Constraint 16 ensures that reactions in $\boldsymbol{J^{kin}}$ are unaffected by the worst-case simulation of the inner problém. Subsequently, we canuse integer cuts (not shown here) and identify all the alternate solutions for κ-interventions. The bilevel problem is converted a single-level Mixed Integer Nonlinear Problem (MINLP) (see the

subsequent section for detailed formulation), which is solved using the BARON solver in GAMS to global optimality.

This optimization problem is solved successively by increasing κ until the target yield is achieved. Given that the objective function of the outer problem is maximization of the product formation, manipulations with the highest impact on the product yield are identified first. By increasing the value of κ additional modifications that improve upon the previously identified ones are revealed thereby providing a way of prioritizing the manipulations based on their impact on the product yield.

Conversion of the single-step bilevel problem into a single level MINLP:

The bilevel problem for identifying the FORCE sets is converted into a single level optimization problem in the following two methods:

a. Using the conditions of Strong Duality

The optimization formulation of the single-level MINLP for the identification of the FORCE sets is as follows:

$$\max \quad v_{prod}$$

$$s.t. \quad \sum_{j \in J^{kin}} y_j^{U,kin} + y_j^{L,kin} + \sum_{j \in J^{stoic}} y_j^{U,stoic} + y_j^{L,stoic} + y_j^{X,stoic} = \kappa, \tag{1}$$

$$y_j^{U,kin} + y_j^{L,kin} \leq 1, \qquad \forall j \in \boldsymbol{J^{kin}} \tag{2}$$

$$y_j^{U,stoic} + y_j^{L,stoic} + y_j^{X,stoic} \leq 1, \qquad \forall j \in \boldsymbol{J^{stoic}} \tag{3}$$

$$r_j = v\left(v_j^{max}, c_i, k_j^p\right), \qquad \forall i \in \boldsymbol{I^{kin}}, j \in \boldsymbol{J^{kin}}, p \in \boldsymbol{P} \tag{4}$$

$$c_i^{min} \leq c_i \leq c_i^{max}, \qquad \forall i \in \boldsymbol{I^{kin}} \tag{5}$$

$$v_j^{max,ref}\left(1 - y_j^{L,kin}\right) \leq v_j^{max} \leq v_j^{max,ref}, \qquad \forall j \in \{\boldsymbol{J^{kin}} \cap \boldsymbol{MUST^L}\} \tag{6}$$

$$v_j^{max,ref} \leq v_j^{max} \leq v_j^{max,ref}\left(1 + (z-1)y_j^{U,kin}\right), \qquad \forall j \in \{\boldsymbol{J^{kin}} \cap \boldsymbol{MUST^U}\} \tag{7}$$

$$v_j^{max} = v_j^{max,ref}, \qquad \forall j \in \{\boldsymbol{J^{kin}} \cap NOT(\boldsymbol{MUST^U} \cup \boldsymbol{MUST^L})\} \tag{8}$$

$$\sum_{j \in J}^{N} S_{ij} v_j = 0, \qquad \forall i \in \boldsymbol{I} \qquad (9)$$

$$LB_j \leq v_j \leq UB_j, \qquad \forall j \in \boldsymbol{J} \qquad (10)$$

$$v_j \geq v_j^{OS,L} + LB_j\left(1 - y_j^{U,stoic}\right), \qquad \forall j \in \{\boldsymbol{J^{stoic}} \cap \boldsymbol{MUST^{U}}\} \qquad (11)$$

$$v_j \leq v_j^{OS,U} + UB_j\left(1 - y_j^{L,stoic}\right), \qquad \forall j \in \{\boldsymbol{J^{stoic}} \cap \boldsymbol{MUST^{L}}\} \qquad (12)$$

$$LB_j\left(1 - y_j^{X,stoic}\right) \leq v_j \leq UB_j\left(1 - y_j^{X,stoic}\right), \qquad \forall j \in \{\boldsymbol{J^{stoic}} \cap \boldsymbol{MUST^{X}}\} \qquad (13)$$

$$v_j = r_j, \qquad \forall j \in \boldsymbol{J^{kin}} \qquad (14)$$

$$\sum_{i \in I} \lambda_i S_{prod} + \gamma_{prod}^{L} - \gamma_{prod}^{U} = 1, \qquad (15)$$

$$\sum_{i \in I} \lambda_i S_{ij} + \delta_j^{U} + \gamma_j^{L} - \gamma_j^{U} = 0, \qquad \forall j \in \{\boldsymbol{J^{stoic}} \cap \boldsymbol{MUST^{U}}\} \qquad (16)$$

$$\sum_{i \in I} \lambda_i S_{ij} - \delta_j^{L} + \gamma_j^{L} - \gamma_j^{U} = 0, \qquad \forall j \in \{\boldsymbol{J^{stoic}} \cap \boldsymbol{MUST^{L}}\} \qquad (17)$$

$$\sum_{i \in I} \lambda_i S_{ij} + \eta_j^{L} - \eta_j^{U} + \gamma_j^{L} - \gamma_j^{U} = 0, \qquad \forall j \in \{\boldsymbol{J^{stoic}} \cap \boldsymbol{MUST^{X}}\} \qquad (18)$$

$$\sum_{i \in I} \lambda_i S_{ij} + \gamma_j^{L} - \gamma_j^{U} = 0, \qquad \forall j \in NOT\{\boldsymbol{prod} \cup \boldsymbol{MUST^{stoic}}\} \qquad (19)$$

$$\sum_{i \in I} \lambda_i S_{ij} + \mu_j + \gamma_j^{L} - \gamma_j^{U} = 0, \qquad \forall j \in \boldsymbol{J^{kin}} \qquad (20)$$

$$\begin{aligned} v_{prod} = \gamma_{prod}^{L} LB_{prod} - \gamma_{prod}^{U} UB_{prod} &+ \sum_{j \in \{J^{stoic} \cap MUST^{U}\}} \delta_j^{U} \left(v_j^{OS,L} + LB_j\left(1 - y_j^{U,stoic}\right)\right) \\ &- \sum_{j \in \{J^{stoic} \cap MUST^{L}\}} \delta_j^{L} \left(v_j^{OS,U} + UB_j\left(1 - y_j^{L,stoic}\right)\right) \\ &+ \sum_{j \in \{J^{stoic} \cap MUST^{X}\}} \eta_j^{L} LB_j\left(1 - y_j^{X,stoic}\right) - \eta_j^{U} UB_j\left(1 - y_j^{X,stoic}\right) \\ &+ \sum_{j \in NOT\{prod \cup MUST^{stoic}\}} \gamma_j^{L} LB_j - \gamma_j^{U} UB_j + \sum_{j \in J^{kin}} \mu_j r_j, \end{aligned} \qquad (21)$$

$$y_j^{U,kin}, y_j^{L,kin}, y_j^{U,stoic}, y_j^{L,stoic}, y_j^{X,stoic} \in \{0,1\}$$

$$\delta_j^{U}, \delta_j^{L}, \eta_j^{U}, \eta_j^{L}, \gamma_j^{U}, \gamma_j^{L} \in \boldsymbol{R}^{+}; \mu_j, \lambda_i \in \boldsymbol{R}$$

Here, the objective function of the problem remains the same as that of the outer problem in the bilevel formulation. In addition, the dual problem of the inner optimization problem (called the primal) is constructed. Each constraint in the primal problem has a corresponding dual variable in the dual problem. Similarly, each constraint of the dual corresponds to a primal variable (i.e. v_j). The signs of the dual variables depend on equality or inequality constraints of the primal, and *vice versa*. δ_j^U and δ_j^L are the dual variables associated with the primal constraints determining the up-regulation (11) and down-regulation (12) of a reaction respectively. η_j^U and η_j^L represent the duals associated with the primal constraints for removal of reactions (13), while μ_j correspond to the fluxes in $\boldsymbol{J^{kin}}$. The duals for the upper and lower bounds of reactions are represented by γ_j^U and γ_j^L respectively. All the constraints of outer level (2-8), inner level (9-14) and its dual problem (15-20) are included in the single-level formulation. The optimization function of the inner problem is replaced by the constraint of strong duality, where the primal (inner) objective set equal to its dual objective (21). This ensures that the inner problem is always optimal whenever we optimize the outer objective.

b. Using the Karush-Kuhn-Tucker (KKT) conditions of optimality

When we enforce the strong condition of duality (Constraint 21) in the previous formulation, we add additional bilinear terms, which increase the nonlinearity of the problem. The nonlinearity arises from the multiplication of the fluxes r_j of the reactions in $\boldsymbol{J^{kin}}$, with their dual variables μ_j. We can avoid these bilinear expressions by replacing the constraint of strong duality (21) of the previous formulation, with the Karush-Kuhn-Tucker (KKT) conditions of optimality:

$$g_j^L = \begin{cases} v_j - v_j^{OS,L} - LB_j\left(1 - y_j^{U,stoic}\right), & \forall j \in \{\boldsymbol{J^{stoic}} \cap \boldsymbol{MUST^U}\} \\ v_j - LB_j\left(1 - y_j^{X,stoic}\right), & \forall j \in \{\boldsymbol{J^{stoic}} \cap \boldsymbol{MUST^X}\} \\ v_j - LB_j, & \forall j \in \left\{\boldsymbol{J^{stoic}} \cap NOT\{\boldsymbol{MUST^{stoic}}\} \cap \boldsymbol{B^L}\right\} \end{cases} \tag{21}$$

$$\alpha_j^L = \begin{cases} \delta_j^U, & \forall j \in \{\boldsymbol{J^{stoic}} \cap \boldsymbol{MUST^U}\} \\ \eta_j^L, & \forall j \in \{\boldsymbol{J^{stoic}} \cap \boldsymbol{MUST^X}\} \\ \gamma_j^L, & \forall j \in \left\{\boldsymbol{J^{stoic}} \cap NOT\{\boldsymbol{MUST^{stoic}}\} \cap \boldsymbol{B^L}\right\} \end{cases} \tag{22}$$

$$g_j^U = \begin{cases} -v_j + v_j^{OS,U} + UB_j\left(1 - y_j^{L,stoic}\right), & \forall j \in \{\boldsymbol{J^{stoic}} \cap \boldsymbol{MUST^L}\} \\ -v_j + UB_j\left(1 - y_j^{X,stoic}\right), & \forall j \in \{\boldsymbol{J^{stoic}} \cap \boldsymbol{MUST^X}\} \\ -v_j + UB_j, & \forall j \in \left\{\boldsymbol{J^{stoic}} \cap NOT\{\boldsymbol{MUST^{stoic}}\} \cap \boldsymbol{B^U}\right\} \end{cases} \tag{23}$$

$$\alpha_j^U = \begin{cases} \delta_j^L, & \forall j \in \{\boldsymbol{J^{stoic}} \cap \boldsymbol{MUST^L}\} \\ \eta_j^U, & \forall j \in \{\boldsymbol{J^{stoic}} \cap \boldsymbol{MUST^X}\} \\ \gamma_j^U, & \forall j \in \{\boldsymbol{J^{stoic}} \cap NOT\{\boldsymbol{MUST^{stoic}}\} \cap \boldsymbol{B^U}\} \end{cases} \quad (24)$$

$$g_j^L \leq g^{max}\left(1 - w_j^L\right), \quad \forall j \in \{\boldsymbol{B^L} \cup \{\boldsymbol{J^{stoic}} \cap \{\boldsymbol{MUST^U} \cup \boldsymbol{MUST^X}\}\}\} \quad (25)$$

$$\alpha_j^L \leq \alpha^{max} w_j^L, \quad \forall j \in \{\boldsymbol{B^L} \cup \{\boldsymbol{J^{stoic}} \cap \{\boldsymbol{MUST^U} \cup \boldsymbol{MUST^X}\}\}\} \quad (26)$$

$$g_j^U \leq g^{max}\left(1 - w_j^U\right), \quad \forall j \in \{\boldsymbol{B^U} \cup \{\boldsymbol{J^{stoic}} \cap \{\boldsymbol{MUST^L} \cup \boldsymbol{MUST^X}\}\}\} \quad (27)$$

$$\alpha_j^U \leq \alpha^{max} w_j^U, \quad \forall j \in \{\boldsymbol{B^U} \cup \{\boldsymbol{J^{stoic}} \cap \{\boldsymbol{MUST^L} \cup \boldsymbol{MUST^X}\}\}\} \quad (28)$$

$$g_j^U, g_j^L, \alpha_j^U, \alpha_j^L \in \boldsymbol{R}^+$$

$$w_j^U, w_j^L \in \{0,1\}$$

Here, g_j^L and g_j^U represent the slacks in the constraints of the primal (inner) problem, while $\alpha_j^L$and $\alpha_j^U$are their corresponding dual variables respectively. The KKT conditions of optimality state that at the optimal point, the primal constraints are slack (i.e. slacks g_j^L and g_j^U are greater than 0) if the dual variables are tight (i.e. $\alpha_j^L$and α_j^U equal 0). Conversely, the primal constraints are tight (i.e. slacks g_j^L and g_j^U are equal 0) when the duals are slack (i.e. $\alpha_j^L$and α_j^U are greater than 0). These conditions are enforced by constraints 25-28. Binary variables $w_j^L$and w_j^U connect the primal slacks to their corresponding dual variables. All other constraints from the previous problem (Constraints 1-20) remain unaltered.

The processing time for the single-step formulation for FORCE sets could be significantly improved if we can pre-estimate the primal constraints that will never be active, and those that will always be inactive. This will allow us to fix the values of some of the slack binary variables (w_j^L , w_j^U) to one or zero. Thestepwise guidelines for identifying (and fixing) some ofw_j^Landw_j^Uvariables areelucidated as follows:

(1) Identify all the blocked reactions in the model, and fix their flux to zero. Subsequently, remove all the dual constraints associated with the blocked reactions.
(2) Formulate the dual problem of the primal inner problem and enforce strong conditions of duality (Constraint 21 of formulation with Strong Duality)

(3) Augment the primal and dual constraints and include all constraints for MUST sets (Constraints 9-20)

(4) Relax all binary variables associated with MUST sets (y_j^{kin} ,y_j^{stoic}) (i.e.,convert them to continuous variable varying in the interval [0-1]).

(5) Minimize the sum of all dual variables (δ_j^U ,η_j^U , γ_j^U ,δ_j^L ,η_j^L , γ_j^L) and identify all dual variables (set A) which are non-zero. The corresponding primal constraints will always be active for these variables.

(6) Maximize the sum of all dual variables (δ_j^U ,η_j^U , γ_j^U ,δ_j^L ,η_j^L , γ_j^L) and identify all dual variables (set B) which are zero. The corresponding primal constraints will be inactive for these variables.

(7) For the formulation with KKT conditions, fix the binary variables (w_j^L,w_j^U) associated with set A variables to one, while fixing the binary variables of set B to zero.

(8) Solve the reduced single-level formulation with KKT conditions to identify the FORCE sets.

2. *Two-step formulation:*

Decomposing the single-step formulation for FORCE sets into two equivalent steps can significantly reduce the computational complexity of solving the problem. The two-step formulation for identifying FORCE sets is described as follows:

Step 1: Identifying minimum set of interventions within $\boldsymbol{J}^{kin}$

The first step minimizes the number of interventions in $\boldsymbol{J}^{kin}$ required to be consistent with desired levels of overproduction of the target chemical.The formulation is as follows:

$$\min \quad \sum_{j \in J^{kin}} y_j^{U,kin} + y_j^{L,kin}$$

$$s.t. \quad y_j^{U,kin} + y_j^{L,kin} \leq 1, \qquad \forall j \in \boldsymbol{J^{kin}} \qquad (1)$$

$$\sum_{j \in J}^{N} S_{ij} v_j = 0, \qquad \forall i \in \boldsymbol{I} \qquad (2)$$

$$u_i = u_i^{upt}, \qquad \forall i \in \boldsymbol{I^{upt}} \qquad (3)$$

$$v_{biomass} \geq v_{biomass}^{min}, v_{prod} \geq v_{pdt}^{target}, \qquad (4)$$

$$LB_j \leq v_j \leq UB_j, \qquad \forall j \in \boldsymbol{J} \qquad (5)$$

$$v_j = v\left(v_j^{max}, c_i, k_j^p\right), \qquad \forall i \in \boldsymbol{I^{kin}}, j \in \boldsymbol{J^{kin}}, p \in \boldsymbol{P} \qquad (6)$$

$$c_i^{min} \leq c_i \leq c_i^{max}, \qquad \forall i \in \boldsymbol{I^{kin}} \qquad (7)$$

$$v_j^{max,ref}\left(1 - y_j^{L,kin}\right) \leq v_j^{max} \leq v_j^{max,ref}, \qquad \forall j \in \{\boldsymbol{J^{kin}} \cap \boldsymbol{MUST^L}\} \qquad (8)$$

$$v_j^{max,ref} \leq v_j^{max} \leq v_j^{max,ref}\left(1 + (z-1) y_j^{U,kin}\right), \qquad \forall j \in \{\boldsymbol{J^{kin}} \cap \boldsymbol{MUST^U}\} \qquad (9)$$

$$v_j^{max} = v_j^{max,ref}, \qquad \forall j \in \{\boldsymbol{J^{kin}} \cap NOT\left(\boldsymbol{MUST^U} \cup \boldsymbol{MUST^L}\right)\} \qquad (10)$$

$$y_j^{U,kin}, y_j^{L,kin} \in \{0,1\}$$

Theconstraints for propagating enzymatic interventions in $\boldsymbol{J^{kin}}$aresame as those enforced in the outer problem for the single-step formulation. The pre-defined levels of production of the desired chemical and biomass are set by Constraint 4. Integer cuts are used to identify all the alternate sets of interventions. This MINLP is solved using the BARON solver in GAMS to global optimality.

Step 2: Identifying additional interventions within $\boldsymbol{J^{stoic}}$:

The optimization formulation for identifying the minimum set of interventions outside the kinetic space to complement Step 1 interventionsis a bilevel problem, described as follows:

$$\max \quad v_{prod}$$

$$s.t. \quad \sum_{j \in J^{stoic}} y_j^{U,stoic} + y_j^{L,stoic} + y_j^{X,stoic} = \kappa, \qquad (1)$$

$$y_j^{U,stoic} + y_j^{L,stoic} + y_j^{X,stoic} \leq 1, \qquad \forall j \in \boldsymbol{J^{stoic}} \qquad (2)$$

$$\min \quad v_{prod}$$

$$\sum_{j \in J}^{N} S_{ij} v_j = 0, \qquad \forall i \in \boldsymbol{I} \qquad (3)$$

$$u_i = u_i^{upt}, \qquad \forall i \in \boldsymbol{I^{uptake}} \qquad (4)$$

$$v_{biomass} \geq v_{biomass}^{min}, \qquad (5)$$

$$LB_j \leq v_j \leq UB_j, \qquad \forall j \in \boldsymbol{J} \qquad (6)$$

$$v_j \geq v_j^{OS,L} + LB_j\left(1 - y_j^{U,stoic}\right), \qquad \forall j \in \{\boldsymbol{J^{stoic}} \cap \boldsymbol{MUST^U}\} \qquad (7)$$

$$v_j \leq v_j^{OS,U} + UB_j\left(1 - y_j^{L,stoic}\right), \qquad \forall j \in \{\boldsymbol{J^{stoic}} \cap \boldsymbol{MUST^L}\} \qquad (8)$$

$$LB_j\left(1 - y_j^{X,stoic}\right) \leq v_j \leq UB_j\left(1 - y_j^{X,stoic}\right), \forall j \in \{\boldsymbol{J^{stoic}} \cap \boldsymbol{MUST^X}\} \qquad (9)$$

$$v_j = v_j^{step1}, \qquad \forall j \in \boldsymbol{J^{kin}} \qquad (10)$$

$$y_j^{U,stoic}, y_j^{L,stoic}, y_j^{X,stoic} \in \{0,1\}$$

Similar to the single-step formulation, the inner optimization problem simulates the “worst-case” scenario for the production of the targeted product while the outer part aims to maximize the overproduction target for a certain number of engineered modifications κin $\boldsymbol{J^{stoic}}$. The constraints propagating these interventions are same as those in the inner problem of single-step formulation. The fluxes in $\boldsymbol{J^{kin}}$ are fixed at their steady-state values $v_j^{step\ 1}$ obtained from the first step of the formulation (10). The number of direct interventions κ in $\boldsymbol{J^{stoic}}$ is successively increased until the target yield is achieved. CPLEX solver in GAMS is used to solve the resulting MILP to global optimum.

Kinetic Model of central metabolism of *E. coli*extracted from Chassagnole*et al*[1]:

All abbreviations for metabolites and reaction names have been imported from the nomenclature of *i*AF1260 model [2] of *E. coli*.

Differential equations for metabolite balances:

$$\frac{dC_{glc-D}^{e}}{dt} = D\left(C_{glc-D}^{feed} - C_{glc-D}^{e}\right) - \frac{C_X v_{GLCptspp}}{\rho_X}$$

$$\frac{dC_{g6p}}{dt} = v_{GLCptspp} - v_{PGI} - v_{G6PDH2} - v_{PGMT} - v_{PGM} - \mu C_{g6p}$$

$$\frac{dC_{f6p}}{dt} = v_{PGI} - v_{PFK} + v_{TKT2} + v_{TALA} - 2v_{MurSynt\ h} - \mu C_{f6p}$$

$$\frac{dC_{fdp}}{dt} = v_{PFK} - v_{FBA} - \mu C_{fdp}$$

$$\frac{dC_{g3p}}{dt} = v_{FBA} + v_{TPI} - v_{GAPD} + v_{TKT1} + v_{TKT2} - v_{TALA} - v_{TrpSynt\ h} - \mu C_{g3p}$$

$$\frac{dC_{dhap}}{dt} = v_{FBA} + v_{TPI} - v_{GAPD} + v_{TKT1} + v_{G3PD} - \mu C_{dhap}$$

$$\frac{dC_{d13dpg}}{dt} = v_{GAPD} - v_{PGK} - \mu C_{13dpg}$$

$$\frac{dC_{3pg}}{dt} = v_{PGK} - v_{PGM} - v_{PGCD} - \mu C_{3pg}$$

$$\frac{dC_{2pg}}{dt} = v_{PGM} - v_{ENO} - \mu C_{2pg}$$

$$\frac{dC_{pep}}{dt} = v_{ENO} - v_{PYK} - v_{GLCptspp} - v_{PPC} - v_{DDPA} - v_{Synt\,h1} - \mu C_{pep}$$

$$\frac{dC_{pyr}}{dt} = v_{PYK} + v_{GLCptspp} - v_{PDH} - v_{Synt\,h2} + v_{MetSynt\ h} + v_{TrpSynt\ h} - \mu C_{pyr}$$

$$\frac{dC_{6pgc}}{dt} = v_{G6PDH} - v_{GND} - \mu C_{6pgc}$$

$$\frac{dC_{ru5p-D}}{dt} = v_{GND} - v_{RPE} - v_{RPI} - \mu C_{ru5p-D}$$

$$\frac{dC_{xu5p-D}}{dt} = v_{RPE} - v_{TKT1} - v_{TKT2} - \mu C_{xu5p-D}$$

$$\frac{dC_{r5p}}{dt} = v_{RPI} - v_{TKT1} - v_{PRPPS} - \mu C_{r5p}$$

$$\frac{dC_{s7p}}{dt} = v_{TKT1} - v_{TALA} - \mu C_{s7p}$$

$$\frac{dC_{e4p}}{dt} = v_{TALA} - v_{TKT2} - v_{DDPA} - \mu C_{e4p}$$

$$\frac{dC_{g1p}}{dt} = v_{PGMT} - v_{GLGC} - v_{DDPA} - \mu C_{g1p}$$

Kinetic rate equations:

$$v_{GLCptspp} = \frac{v_{GLCptspp}^{max}\, C_{glc-D}^{e} \frac{C_{pep}}{C_{pyr}}}{\left(K_{PTS,a1} + K_{PTS,a2}\frac{C_{pep}}{C_{pyr}} + K_{PTS,a3} C_{glc-D}^{e} + C_{glc-D}^{e}\frac{C_{pep}}{C_{pyr}}\right)\left(1 + C_{g6p}^{n_{PTS,g6p}}\right)}$$

$$v_{PGI} = \frac{v_{PGI}^{max}\left(C_{g6p} - \frac{C_{f6p}}{K_{PGI,eq}}\right)}{K_{PGI,g6p}\left(1 + \frac{C_{f6p}}{K_{PGI,f6p}\left(1+\frac{C_{6pgc}}{K_{PGI,f6p,6pgcinh}}\right)} + \frac{C_{6pgc}}{K_{PGI,g6p,6pgcinh}}\right) + C_{g6p}}$$

$$v_{PFK} = \frac{v_{PFK}^{max}\, C_{atp}\, C_{f6p}}{\left(C_{atp} + K_{PFK,atp,s}\left(1 + \frac{C_{adp}}{K_{PFK,adp,c}}\right)\right)\left(C_{f6p} + K_{PFK,f6p,s}\frac{A}{B}\right)\left(1 + \frac{L_{PFK}}{\left(1+C_{f6p}\frac{B}{K_{PFK,f6p,s}A}\right)^{n_{PFK}}}\right)}$$

$$A = 1 + \frac{C_{pep}}{K_{PFK,pep}} + \frac{C_{adp}}{K_{PFK,adp,b}} + \frac{C_{amp}}{K_{PFK,amp,b}}$$

$$B = 1 + \frac{C_{adp}}{K_{PFK,adp,a}} + \frac{C_{amp}}{K_{PFK,amp,a}}$$

$$v_{FBA} = \frac{v_{FBA}^{max}\left(C_{fdp} - \frac{C_{g3p} C_{dhap}}{K_{FBA,eq}}\right)}{K_{FBA,fdp} + C_{fdp} + \frac{K_{FBA,g3p} C_{dhap}}{K_{FBA,eq} r_{ALDO,blf}} + \frac{K_{FBA,dhap} C_{g3p}}{K_{FBA,eq} r_{ALDO,blf}} + \frac{C_{g3p} C_{fdp}}{K_{FBA,g3p,inh}} + \frac{C_{g3p} C_{dhap}}{K_{FBA,eq} r_{ALDO,blf}}}$$

$$v_{TPI} = \frac{v_{TPI}^{max}\left(C_{dhap} - \frac{C_{g3p}}{K_{TPI,eq}}\right)}{K_{TPI,dhap}\left(1 + \frac{C_{g3p}}{K_{TPI,g3p}}\right) + C_{dhap}}$$

$$v_{GAPD} = \frac{v_{GAPD}^{max}\left(C_{g3p}C_{nad} - \frac{C_{13dpg}C_{nad\,h}}{K_{GAPD\,,eq}}\right)}{\left(K_{GAPD,g3p}\left(1+\frac{C_{13dpg}}{K_{GAPD\,,13dpg}}\right)+C_{g3p}\right)\left(K_{GAPD,nad}\left(1+\frac{C_{nad\,h}}{K_{GAPD\,,nad\,h}}\right)+C_{nad}\right)}$$

$$v_{PGK} = \frac{v_{PGK}^{max}\left(C_{adp}C_{13dpg} - \frac{C_{atp}C_{3pg}}{K_{PGK\,,eq}}\right)}{\left(K_{PGK,adp}\left(1+\frac{C_{atp}}{K_{PGK\,,atp}}\right)+C_{adp}\right)\left(K_{PGK,13dpg}\left(1+\frac{C_{3pg}}{K_{PGK\,,3pg}}\right)+C_{13dpg}\right)}$$

$$v_{PGM} = \frac{v_{PGM}^{max}\left(C_{3pg} - \frac{C_{2pg}}{K_{PGM\,,eq}}\right)}{K_{PGM,3pg}\left(1+\frac{C_{2pg}}{K_{PGM\,,2pg}}\right)+C_{3pg}}$$

$$v_{ENO} = \frac{v_{ENO}^{max}\left(C_{2pg} - \frac{C_{pep}}{K_{ENO\,,eq}}\right)}{K_{ENO,2pg}\left(1+\frac{C_{pep}}{K_{ENO\,,pep}}\right)+C_{2pg}}$$

$$v_{PYK} = \frac{v_{PYK}^{max}C_{pep}\left(\frac{C_{pep}}{K_{PYK\,,pep}}+1\right)^{(n_{PYK}-1)}C_{adp}}{K_{PYK,pep}\left(L_{PYK}\left(\frac{1+\frac{C_{atp}}{K_{PYK\,,atp}}}{\frac{C_{fdp}}{K_{PYK\,,fdp}}+\frac{C_{amp}}{K_{PYK\,,amp}}+1}\right)^{n_{PYK}}+\left(\frac{C_{pep}}{K_{PYK\,,pep}}+1\right)^{n_{PYK}}\right)\left(C_{adp}+K_{PYK,adp}\right)}$$

$$v_{PDH} = \frac{v_{PDH}^{max}C_{pyr}^{n_{PDH}}}{K_{PDH,pyr}+C_{pyr}^{n_{PDH}}}$$

$$v_{PPC} = \frac{v_{PPC}^{max}C_{pep}\left(1+\left(\frac{C_{fdp}}{K_{PPC\,,fdp}}\right)^{n_{PPC\,,fdp}}\right)}{K_{PPC,pep}+C_{pep}}$$

$$v_{PGMT} = \frac{v_{PGMT}^{max}\left(C_{g6p} - \frac{C_{g1p}}{K_{PGMT\,,eq}}\right)}{K_{PGMT,g6p}\left(1+\frac{C_{g1p}}{K_{PGMT\,,g1p}}\right)+C_{g6p}}$$

$$v_{GLGC} = \frac{v_{GLGC}^{max}C_{g1p}C_{atp}\left(1+\left(\frac{C_{fdp}}{K_{GLGC\,,fdp}}\right)^{n_{GLGC}}\right)}{\left(K_{GLGC,g1p}+C_{g1p}\right)\left(K_{GLGC,atp}+C_{atp}\right)}$$

$$v_{PRPPS} = \frac{v_{PRPPS}^{max}C_{r5p}}{K_{PRPPS,pyr}+C_{r5p}}$$

$$v_{G3PD} = \frac{v_{G3PD}^{max} C_{dhap}}{K_{G3PD,dhap} + C_{dhap}}$$

$$v_{PGCD} = \frac{v_{PGCD}^{max} C_{3pg}}{K_{PGCD,3pg} + C_{3pg}}$$

$$v_{MurSynt\ h} = v_{MurSynt\ h}^{max}$$

$$v_{DDPA} = \frac{v_{DDPA}^{max} C_{e4p}^{n_{DDPA,e4p}} C_{pep}^{n_{DDPA,pep}}}{\left(K_{DDPA,e4p} + C_{e4p}^{n_{DDPA,e4p}}\right)\left(K_{DDPA,pep} + C_{pep}^{n_{DDPA,pep}}\right)}$$

$$v_{TrpSynt\ h} = v_{TrpSynt\ h}^{max}$$

$$v_{MetSynt\ h} = v_{MetSynt\ h}^{max}$$

$$v_{G6PDH} = \frac{v_{G6PDH}^{max} C_{g6p} C_{nadp}}{\left(C_{g6p} + K_{G6PDH,g6p}\right)\left(1 + \frac{C_{nadp\ h}}{K_{G6PDH,nadp\ h,g6pin\ h}}\right)\left(K_{G6PDH,nadp}\left(1 + \frac{C_{nadp\ h}}{K_{G6PDH,nadp\ h,nadpin\ h}}\right) + C_{nadp}\right)}$$

$$v_{GND} = \frac{v_{GND}^{max} C_{6pgc} C_{nadp}}{\left(C_{6pgc} + K_{GND,6pgc}\right)\left(C_{nadp} + K_{GND,nadp}\left(1 + \frac{C_{nadp\ h}}{K_{GND,nadp\ h,in\,h}}\right)\left(1 + \frac{C_{atp}}{K_{GND,atp,in\,h}}\right)\right)}$$

$$v_{RPE} = v_{RPE}^{max}\left(C_{ru5p-D} - \frac{C_{xu5p-D}}{K_{RPE,eq}}\right)$$

$$v_{RPI} = v_{RPI}^{max}\left(C_{ru5p-D} - \frac{C_{r5p}}{K_{RPI,eq}}\right)$$

$$v_{TKT1} = v_{TKT1}^{max}\left(C_{r5p} C_{xu5p-D} - \frac{C_{s7p} C_{g3p}}{K_{TKT1,eq}}\right)$$

$$v_{TALA} = v_{TALA}^{max}\left(C_{s7p} C_{g3p} - \frac{C_{e4p} C_{f6p}}{K_{TALA,eq}}\right)$$

$$v_{TKT2} = v_{TKT2}^{max}\left(C_{e4p} C_{xu5p-D} - \frac{C_{f6p} C_{g3p}}{K_{TKT2,eq}}\right)$$

$$v_{Synt\ h1} = \frac{v_{Synt\ h1}^{max} C_{pep}}{K_{Synt\ h1,pep} + C_{pep}}$$

$$v_{Synt\ h2} = \frac{v_{Synt\ h2}^{max} C_{pyr}}{K_{Synt\ h2,pyr} + C_{pyr}}$$

Kinetic Parameters:

Parameter	Value	Unit	Parameter	Value	Unit	Parameter	Value	Unit
$K_{PTS,a1}$	3082.3	mM	$K_{GAPD,13dpg}$	1.04e-5	mM	$K_{GLGC,g1p}$	3.2	mM
$K_{PTS,a2}$	0.01	mM	$K_{GAPD,nad}$	0.252	mM	$K_{GLGC,atp}$	4.42	mM
$K_{PTS,a3}$	245.3	mM	$K_{GAPD,nadh}$	1.09	mM	n_{GLGC}	1.2	
$n_{PTS,g6p}$	3.66		$K_{PGK,eq}$	1934.4		$K_{PRPPS,pyr}$	0.1	mM
$K_{PGI,eq}$	0.1725		$K_{PGK,adp}$	0.185	mM	$K_{G3PD,dhap}$	1	mM
$K_{PGI,g6p}$	2.9	mM	$K_{PGK,atp}$	0.653	mM	$K_{PGCD,3pg}$	1	mM
$K_{PGI,f6p}$	0.266	mM	$K_{PGK,13dpg}$	0.0468	mM	$v_{MurSynth}^{max}$	-0.013	
$K_{PGI,f6p,6pgcinh}$	0.2	mM	$K_{PGK,3pg}$	0.473	mM	$K_{DDPA,e4p}$	0.035	mM
$K_{PGI,g6p,6pgcinh}$	0.2	mM	$K_{PGM,eq}$	0.188		$K_{DDPA,pep}$	0.0053	mM
$K_{PFK,atp,s}$	0.123	mM	$K_{PGM,3pg}$	0.2	mM	$n_{DDPA,e4p}$	2.6	
$K_{PFK,adp,a}$	128	mM	$K_{PGM,2pg}$	0.369	mM	$n_{DDPA,pep}$	2.2	mM
$K_{PFK,adp,b}$	3.89	mM	$K_{ENO,eq}$	6.73		$v_{TrpSynth}^{max}$	0.134	
$K_{PFK,adp,c}$	4.14	mM	$K_{ENO,2pg}$	0.1	mM	$v_{MetSynth}^{max}$	0.134	
$K_{PFK,amp,a}$	19.1	mM	$K_{ENO,pep}$	0.135	mM	$K_{G6PDH,g6p}$	14.4	mM
$K_{PFK,amp,b}$	3.2	mM	$K_{PYK,pep}$	0.31	mM	$K_{G6PDH,nadph,g6pinh}$	6.43	mM
$K_{PFK,f6p,s}$	0.325	mM	L_{PYK}	1000		$K_{G6PDH,nadp}$	0.0246	mM
L_{PFK}	5629067		$K_{PYK,atp}$	22.5	mM	$K_{G6PDH,nadph,nadpinh}$	0.01	mM
n_{PFK}	11.1		$K_{PYK,adp}$	0.26	mM	$K_{GND,6pgc}$	37.5	mM
$K_{PFK,pep}$	3.26	mM	$K_{PYK,fdp}$	0.19	mM	$K_{GND,nadp}$	0.0506	mM
$K_{FBA,eq}$	0.144		$K_{PYK,amp}$	0.2	mM	$K_{GND,nadph,inh}$	0.0138	mM
$K_{FBA,fdp}$	1.75	mM	n_{PYK}	4		$K_{GND,atp,inh}$	208	mM
$K_{FBA,g3p}$	0.088	mM	$K_{PDH,pyr}$	1159	mM	$K_{RPE,eq}$	1.4	
$r_{ALDO,blf}$	2		n_{PDH}	3.68		$K_{RPI,eq}$	4	
$K_{FBA,dhap}$	0.088	mM	$K_{PPC,fdp}$	0.7	mM	$K_{TKT1,eq}$	1.2	
$K_{FBA,g3p,inh}$	0.6	mM	$K_{PPC,pep}$	4.07	mM	$K_{TALA,eq}$	1.05	
$K_{TPI,eq}$	1.39		$n_{PPC,fdp}$	4.21	mM	$K_{TKT2,eq}$	10	
$K_{TPI,dhap}$	2.8	mM	$K_{PGMT,eq}$	0.196		$K_{Synth1,pyr}$	1	mM
$K_{TPI,g3p}$	0.3	mM	$K_{PGMT,g6p}$	1.038	mM	$K_{Synth2,pyr}$	1	mM
$K_{GAPD,eq}$	0.63		$K_{PGMT,g1p}$	0.0136	mM			
$K_{GAPD,g3p}$	0.683	mM	$K_{GLGC,fdp}$	0.119	mM			

v^{max} of reactions (in mmol gDW^{-1} hr^{-1}):

Parameter	Value	Parameter	Value	Parameter	Value
$v_{GLCptspp}^{max}$	2.5404e08	v_{PYK}^{max}	30.50	v_{G6PDH}^{max}	693.13
v_{PGI}^{max}	323155.53	v_{PDH}^{max}	2997.05	v_{GND}^{max}	8148.96
v_{PFK}^{max}	924893.62	v_{PPC}^{max}	62.00	v_{RPE}^{max}	3379.28
v_{FBA}^{max}	8750.234	v_{PGMT}^{max}	332.68	v_{RPI}^{max}	2436.57
v_{TPI}^{max}	34389.38	v_{GLGC}^{max}	2.88	v_{TKT1}^{max}	4803.26
v_{GAPD}^{max}	460441.92	v_{PRPPS}^{max}	6.293	v_{TALA}^{max}	5510.94
v_{PGK}^{max}	1451948.94	v_{G3PD}^{max}	7.47	v_{TKT2}^{max}	42840.70
v_{PGM}^{max}	50203.72	v_{PGCD}^{max}	13.117	$v_{Synt\,h1}^{max}$	4.00
v_{ENO}^{max}	164840.43	v_{DDPA}^{max}	61.92	$v_{Synt\,h2}^{max}$	36.08

Initial metabolite concentrations (in mM):

Metabolite	Value	Metabolite	Value	Metabolite	Value
C_{glc-D}^{e}	2	C_{d13dpg}	0.008	C_{ru5p-D}	0.111
C_{g6p}	3.48	C_{3pg}	2.13	C_{xu5p-D}	0.138
C_{f6p}	0.6	C_{2pg}	0.399	C_{r5p}	0.398
C_{fdp}	0.272	C_{pep}	2.67	C_{s7p}	0.276
C_{g3p}	0.218	C_{pyr}	2.67	C_{e4p}	0.098
C_{dhap}	0.167	C_{6pgc}	0.808	C_{g1p}	0.653

Fixed metabolite concentrations (in mM):

Metabolite	Value	Metabolite	Value
C_{glc-D}^{feed}	50	C_{nad}	1.314
C_{atp}	4.16	$C_{nad\,h}$	0.0934
C_{adp}	0.595	C_{nadp}	0.1588
C_{amp}	0.165	$C_{nadp\,h}$	0.062

Other parameters:

Parameter	Value	Unit	Parameter	Value	Unit
μ	0.1	hr^{-1}	ρ_X	564	gDW hr^{-1}
D	0.1	hr^{-1}	a	1	
C_X	8.7	gDW hr^{-1}			

Kinetic Model of central metabolism of *S. cerevisiae* constructed from van Eunen*et al*[3]and Gombert*et al*[4]:

Differential equations for metabolite balances:

$$\frac{dC_{glc-D}}{dt} = v_{GLCt} - v_{HEX1} + 2\, v_{TREH}$$

$$\frac{dC_{g6p}}{dt} = v_{HEX1} - v_{PGI} - v_{G6PDH2} - v_{TRE6PS}$$

$$\frac{dC_{f6p}}{dt} = v_{PGI} - v_{PFK} + v_{TKT2} + v_{TALA}$$

$$\frac{dC_{fdp}}{dt} = v_{PFK} - v_{FBA}$$

$$\frac{dC_{g3p}}{dt} = 2\, v_{FBA} - v_{GAPD} - v_{G3PT} + v_{TKT1} + v_{TKT2} - v_{TALA}$$

$$\frac{dC_{d13dpg}}{dt} = v_{GAPD} - v_{PGK}$$

$$\frac{dC_{3pg}}{dt} = v_{PGK} - v_{PGM} - v_{PGCD}$$

$$\frac{dC_{2pg}}{dt} = v_{PGM} - v_{ENO}$$

$$\frac{dC_{pep}}{dt} = v_{ENO} - v_{PYK}$$

$$\frac{dC_{pyr}}{dt} = v_{PYK} - v_{PYRDC} - 2 * v_{SUCC}$$

$$\frac{dC_{acald}}{dt} = v_{PYRDC} - v_{ALCD} - 2 * v_{PYRT2m} - v_{ALDD}$$

$$\frac{dC_{nad\,h}}{dt} = v_{GAPD} + v_{G3PT} - v_{ALCD} + 3 * v_{PYRT2m} + v_{ALDD}$$

The following reactions were added to the kinetic model using MFA information of central metabolism of *S. cerevisiae*fromGombert*et al*[4] to account for biomass production:

1. Reactions in Pentose Phosphate Pathway (G6PDH, TKT1, TALA and TKT2)
2. Flux towards L-serine and glycine synthesis (PGCD)

Kinetic rate equations:

$$v_{GLCt} = \frac{v_{GLCt}^{max}\left(\frac{C_{glc-D}^{e}}{K_{GLCt,glce}^{m}} - \frac{C_{glc-D}}{K_{GLCt,glc}^{m} K_{GLCt}^{eq}}\right)}{1 + \frac{C_{glc-D}^{e}}{K_{GLCt,glce}^{m}} + \frac{C_{glc-D}}{K_{GLCt,glci}^{m}} + 0.91\frac{C_{glc-D}^{e} C_{glc-D}}{K_{GLCt,glce}^{m} K_{GLCt,glci}^{m}}}$$

$$v_{HEX1} = \frac{v_{HEX1}^{max}\left(\frac{C_{glc-D}}{K_{HEX\,1,glci}^{m}}\frac{C_{atp}}{K_{HEX\,1,atp}^{m}} - \frac{C_{g6p} C_{adp}}{K_{HEX\,1,atp}^{m} K_{HEX\,1,glci}^{m} K_{HEX\,1}^{eq}}\right)}{\left(1 + \frac{C_{glc-D}}{K_{HEX\,1,glci}^{m}} + \frac{C_{g6p}}{K_{HEX\,1,g6p}^{m}} + \frac{C_{tre\,6p}}{K_{HEX\,1,tre\,6p}^{i}}\right)\left(1 + \frac{C_{atp}}{K_{HEX\,1,atp}^{m}} + \frac{C_{adp}}{K_{HEX\,1,adp}^{m}}\right)}$$

$$v_{TREH} = K_{TREH}$$

$$v_{TRE6PS} = K_{TRE6PS}$$

$$v_{G6PDH} = K_{G6PDH}$$

$$v_{TKT1} = K_{TKT1}$$

$$v_{TKT2} = K_{TKT2}$$

$$v_{TALA} = K_{TALA}$$

$$v_{G3PT} = K_{G3PT}$$

$$v_{PGCD} = K_{PGCD}$$

$$v_{PGI} = \frac{v_{PGI}^{max}\left(\frac{C_{g6p}}{K_{PGI,g6p}^{m}} - \frac{C_{f6p}}{K_{PGI,f6p}^{m} K_{PGI}^{eq}}\right)}{1 + \frac{C_{g6p}}{K_{PGI,g6p}^{m}} - \frac{C_{f6p}}{K_{PGI,f6p}^{m}}}$$

$$v_{PFK} = \frac{A}{B},$$

$$A = v_{PFK}^{max}\; gR\, C_{atp}\, C_{f6p}\left(1 + \frac{C_{f6p}}{K_{PFK,f6p}^{m}}\frac{C_{atp}}{K_{PFK,atp}^{m}} + \frac{gR\, C_{atp}\, C_{f6p}}{K_{PFK,f6p}^{m} K_{PFK,atp}^{m}}\right)$$

$$B = K^m_{PFK,f6p} K^m_{PFK,atp} \left(\left(1 + \frac{C_{f6p}}{K^m_{PFK,f6p}} \frac{C_{atp}}{K^m_{PFK,atp}} + \frac{gR\, C_{atp}\, C_{f6p}}{K^m_{PFK,f6p} K^m_{PFK,atp}}\right)^2 + \left(\frac{L_{PFK}\left(1 + \frac{C^i_{PFK,atp}\, C_{atp}}{K^i_{PFK,atp}}\right)^2 \left(1 + \frac{C_{PFK,f26dp}\, C_{f26bp}}{K_{PFK,f26dp}} + \frac{C_{PFK,fdp}\, C_{fdp}}{K_{PFK,fdp}}\right)^2}{\left(1 + \frac{C_{atp}}{K^i_{PFK,atp}}\right)^2 \left(1 + \frac{C_{amp}}{K^i_{PFK,amp}}\right)^2 \left(1 + \frac{C_{f26bp}}{K_{PFK,f26dp}} + \frac{C_{fdp}}{K_{PFK,fdp}}\right)^2} \right) * \left(1 + \frac{C^i_{PFK,atp}\, C_{atp}}{K^i_{PFK,atp}}\right)^2 \right)$$

$$v_{FBA} = \frac{v^{max}_{FBA} \left(\frac{C_{fdp}}{K^m_{FBA,fdp}} - \frac{\frac{K^{eq}_{TPI}}{1+K^{eq}_{TPI}} C_{g3p} \frac{1}{1+K^{eq}_{TPI}} C_{g3p}}{K^m_{FBA,fdp} K^{eq}_{FBA}} \right)}{1 + \frac{C_{fdp}}{K^m_{FBA,fdp}} + \frac{\frac{K^{eq}_{TPI}}{1+K^{eq}_{TPI}} C_{g3p}}{K^m_{FBA,g3p}} + \frac{\frac{1}{1+K^{eq}_{TPI}} C_{g3p}}{K^m_{FBA,dhap}} + \frac{\frac{K^{eq}_{TPI}}{1+K^{eq}_{TPI}} C_{g3p} \frac{1}{1+K^{eq}_{TPI}} C_{g3p}}{K^m_{FBA,g3p} K^m_{FBA,dhap}} + \frac{\frac{K^{eq}_{TPI}}{1+K^{eq}_{TPI}} C_{g3p}\, C_{fdp}}{K^m_{FBA,fdp} K^m_{FBA,g3pi}}}$$

$$v_{GAPD} = \frac{C_{GAPD} \left(\frac{v^{max}_{GAPDf} \frac{K^{eq}_{TPI}}{1+K^{eq}_{TPI}} C_{g3p} (C_{nad} - C_{nadh})}{K^m_{GAPD,g3p} K^m_{GAPD,nad}} - \frac{v^{max}_{GAPDr}\, C_{nadh} C_{13dpg}}{K^m_{GAPD,13dpg} K^m_{GAPD,nadh}} \right)}{\left(1 + \frac{\frac{K^{eq}_{TPI}}{1+K^{eq}_{TPI}} C_{g3p}}{K^m_{GAPD,g3p}} + \frac{C_{13dpg}}{K^m_{GAPD,nadh}}\right) \left(1 + \frac{C_{nad} - C_{nadh}}{K^m_{GAPD,nad}} + \frac{C_{nadh}}{K^m_{GAPD,nadh}}\right)}$$

$$v_{PGK} = \frac{v^{max}_{PGK} \left(\frac{K^{eq}_{PGK}\, C_{13dpg}\, C_{adp}}{K^m_{PGK,3pg} K^m_{PGK,atp}} - \frac{C_{3pg}\, C_{atp}}{K^m_{PGK,3pg} K^m_{PGK,atp}} \right)}{\left(1 + \frac{C_{13dpg}}{K^m_{PGK,13dpg}} + \frac{C_{3pg}}{K^m_{PGK,3pg}}\right) \left(1 + \frac{C_{atp}}{K^m_{PGK,atp}} + \frac{C_{adp}}{K^m_{PGK,adp}}\right)}$$

$$v_{PGM} = \frac{v^{max}_{PGM} \left(\frac{C_{3pg}}{K^m_{PGM,3pg}} - \frac{C_{2pg}}{K^m_{PGM,3pg} K^{eq}_{PGM}} \right)}{1 + \frac{C_{2pg}}{K^m_{PGM,2pg}} + \frac{C_{3pg}}{K^m_{PGM,3pg}}}$$

$$v_{ENO} = \frac{v_{ENO}^{max}\left(\frac{C_{2pg}}{K_{ENO,2pg}^{m}} - \frac{C_{pep}}{K_{ENO,2pg}^{m}K_{ENO}^{eq}}\right)}{1 + \frac{C_{2pg}}{K_{ENO,2pg}^{m}} + \frac{C_{pep}}{K_{ENO,pep}^{m}}}$$

$$v_{PYK} = \frac{v_{PYK}^{max}\frac{C_{pep}}{K_{PYK,pep}^{m}}\left(1 + \frac{C_{pep}}{K_{PYK,pep}^{m}}\right)^{(n_{PYK}-1)} C_{adp}}{\left(L_{PYK}\left(\frac{1+\frac{C_{atp}}{K_{PYK,atp}^{m}}}{1+\frac{C_{fdp}}{K_{PYK,fdp}^{m}}}\right)^{n_{PYK}} + \left(1 + \frac{C_{pep}}{K_{PYK,pep}^{m}}\right)^{n_{PYK}}\right)\left(C_{adp} + K_{PYK,adp}^{m}\right)}$$

$$v_{PYRDC} = \frac{v_{PYRDC}^{max}C_{pyr}^{n_{PYRDC}}}{{K_{PYRDC,pyr}^{m}}^{n_{PYRDC}}\left(1 + \left(\frac{C_{pep}}{K_{PYK,pep}^{m}}\right)^{n_{PYRDC}}\right)}$$

$$v_{ALCD} = \frac{M}{N},$$

$$M = -v_{ALCD}^{max}\left(\frac{(C_{nad} - C_{nad\,h})C_{eto\,h}}{K_{ALCD,nad}^{i}K_{ALCD,eto\,h}^{m}} - \frac{C_{nad\,h}C_{acald}}{K_{ALCD,nad}^{i}K_{ALCD,eto\,h}^{m}K_{ALCD}^{eq}}\right)$$

$$\begin{aligned} N = 1 &+ \frac{C_{nad} - C_{nad\,h}}{K_{ALCD,nad}^{i}} + \frac{K_{ALCD,nad}^{m}C_{eto\,h}}{K_{ALCD,nad}^{i}K_{ALCD,eto\,h}^{m}} + \frac{C_{nad\,h}C_{acald}}{K_{ALCD,nad\,h}^{i}K_{ALCD,acald}^{m}} + \frac{C_{nad\,h}}{K_{ALCD,nad\,h}^{i}} \\ &+ \frac{(C_{nad} - C_{nad\,h})C_{eto\,h}}{K_{ALCD,nad}^{i}K_{ALCD,eto\,h}^{m}} + \frac{K_{ALCD,nad\,h}^{m}(C_{nad} - C_{nad\,h})C_{acald}}{K_{ALCD,nad}^{i}K_{ALCD,nad\,h}^{i}K_{ALCDacald}^{m}} \\ &+ \frac{K_{ALCD,nad}^{m}C_{eto\,h}C_{nad\,h}}{K_{ALCD,nad}^{i}K_{ALCD,eto\,h}^{m}K_{ALCD,nad\,h}^{i}} + \frac{C_{acald}C_{nad\,h}}{K_{ALCD,nad\,h}^{i}K_{ALCD,acald}^{m}} \\ &+ \frac{(C_{nad} - C_{nad\,h})C_{acald}C_{eto\,h}}{K_{ALCD,nad}^{i}K_{ALCD,eto\,h}^{m}K_{ALCDacald}^{i}} + \frac{C_{eto\,h}C_{nad\,h}C_{acald}}{K_{ALCD,nad\,h}^{i}K_{ALCD,eto\,h}^{i}K_{ALCD,acald}^{m}} \end{aligned}$$

$$v_{PYRt2m} = K_{PYRt2m}$$

$$v_{ALDD} = K_{ALDD}C_{acald}$$

Kinetic Parameters:

Parameter	Value	Unit	Parameter	Value	Unit	Parameter	Value	Unit
$K^{m}_{GLCt,glce}$	1.6	mM	$K^{m}_{FBA,fdp}$	0.3	mM	$K^{m}_{PYK,adp}$	0.3	mM
$K^{m}_{GLCt,glci}$	1.6	mM	$K^{m}_{FBA,g3p}$	2	mM	L_{PYK}	60000	
K^{eq}_{GLCt}	1		$K^{m}_{FBA,dhap}$	2.4	mM	n_{PYK}	4	
$K^{m}_{HEX1,glci}$	0.08	mM	$K^{m}_{FBA,g3pi}$	10	mM	$K^{m}_{PYRDC,pyr}$	6.36	mM
$K^{m}_{HEX1,atp}$	0.15	mM	K^{eq}_{TPI}	0.045		n_{PYRDC}	1.9	
$K^{m}_{HEX1,adp}$	0.23	mM	K^{eq}_{FBA}	0.069		$K^{m}_{ALCD,nad}$	0.17	mM
$K^{m}_{HEX1,g6p}$	30	mM	$K^{m}_{GAPD,g3p}$	0.39	mM	$K^{i}_{ALCD,nad}$	0.92	mM
$K^{i}_{HEX1,tre6p}$	0.04	mM	$K^{m}_{GAPD,13dpg}$	0.51	mM	$K^{m}_{ALCD,nadh}$	0.11	mM
K^{eq}_{HEX1}	3800		$K^{m}_{GAPD,nad}$	2.85	mM	$K^{i}_{ALCD,nadh}$	0.031	mM
$K^{m}_{PGI,g6p}$	1.4	mM	$K^{m}_{GAPD,nadh}$	0.007	mM	$K^{m}_{ALCD,etoh}$	17	mM
$K^{m}_{PGI,f6p}$	0.3	mM	C_{GAPD}	1	mM	$K^{i}_{ALCD,etoh}$	90	mM
K^{eq}_{PGI}	0.314		$K^{m}_{PGK,3pg}$	0.53	mM	$K^{m}_{ALCD,acald}$	1.11	mM
gR	5.12		$K^{m}_{PGK,13dpg}$	0.003	mM	$K^{i}_{ALCD,acald}$	1.1	mM
L_{PFK}	0.66		$K^{m}_{PGK,adp}$	0.2	mM	K^{eq}_{ALCD}	6.9e-5	
$K^{m}_{PFK,f6p}$	0.1	mM	$K^{m}_{PGK,atp}$	0.3	mM	K_{TREH}	0	mM min^{-1}
$K^{m}_{PFK,atp}$	0.71	mM	K^{eq}_{PGK}	3200		K_{TRE6PS}	2.45	mM min^{-1}
$C^{i}_{PFK,atp}$	100		$K^{m}_{PGM,3pg}$	1.2	mM	K_{G3PT}	18.34	mM min^{-1}
$C_{PFK,atp}$	3		$K^{m}_{PGM,2pg}$	0.08	mM	K_{ALDD}	0.59	mM min^{-1}
$C_{PFK,amp}$	0.0845		K^{eq}_{PGM}	0.19		K_{PYRt2m}	2.34	mM min^{-1}
$C_{PFK,f26bp}$	0.0174		$K^{m}_{ENO,2pg}$	0.04	mM	K_{G6PDH}	20	mM min^{-1}
$C_{PFK,fdp}$	0.397		$K^{m}_{ENO,pep}$	0.5	mM	K_{TKT1}	6.66	mM min^{-1}
$K^{i}_{PFK,atp}$	0.65	mM	K^{eq}_{ENO}	6.7		K_{TALA}	6.66	mM min^{-1}
$K^{i}_{PFK,amp}$	0.0995	mM	$K^{m}_{PYK,pep}$	0.19	mM	K_{TKT2}	6.13	mM min^{-1}
$K_{PFK,f26dp}$	0.000682	mM	$K^{m}_{PYK,fdp}$	0.2	mM	K_{PGCD}	0.78	mM min^{-1}
$K_{PFK,fdp}$	0.111	mM	$K^{m}_{PYK,atp}$	9.3	mM			

v^{max} of reactions (in mM min^{-1}):

Parameter	Value	Parameter	Value	Parameter	Value
v_{GLCt}^{max}	256.88	v_{GAPDf}^{max}	2440.37	v_{PYK}^{max}	652.71
v_{HEX1}^{max}	332.78	v_{GAPDr}^{max}	1414.01	v_{PYRDC}^{max}	289.58
v_{PGI}^{max}	943.46	v_{PGK}^{max}	3117.61	v_{ALCD}^{max}	953.96
v_{PFK}^{max}	248.7	v_{PGM}^{max}	999.50		
v_{FBA}^{max}	220.69	v_{ENO}^{max}	416.85		

Initial metabolite concentrations (in mM):

Metabolite	Value	Metabolite	Value	Metabolite	Value
C_{glc-D}	0.1	C_{g3p}	1	C_{pep}	0.1
C_{g6p}	3.8	C_{d13dpg}	0.00001	C_{pyr}	2.8
C_{f6p}	0.74	C_{3pg}	0.69	C_{acald}	0.04
C_{fdp}	12	C_{2pg}	0.09	C_{nadh}	0.29

Fixed metabolite concentrations (in mM):

Metabolite	Value	Metabolite	Value	Metabolite	Value
C_{nad}	1.59	C_{f26bp}	0.014	C_{treh}	2
C_{atp}	5	C_{co2}	1	C_{glc-D}^{e}	50
C_{adp}	1	C_{etoh}	25	C_{glyc}	10
C_{amp}	0.3	C_{tre6p}	2.2	C_{ac}	10

Supplementary Figures:

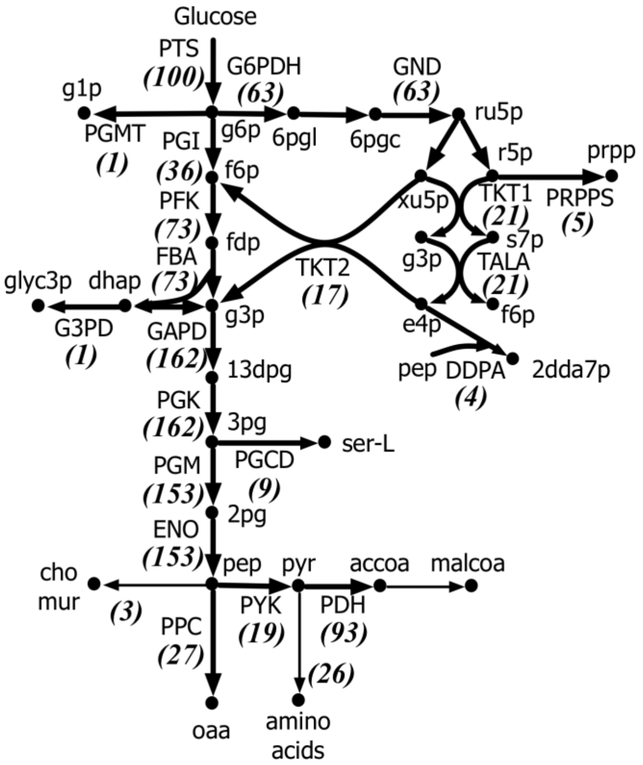

Figure S1: The steady-state distribution of fluxes in the central carbon metabolism of *E. coli* obtained after solving the system of ODEs for the kinetic model proposed in Chassagnole *et al* [1]. Values in brackets indicate the flux in mmol $gDW^{-1}hr^{-1}$ per 100 mmol $gDW^{-1}hr^{-1}$ of glucose uptake.

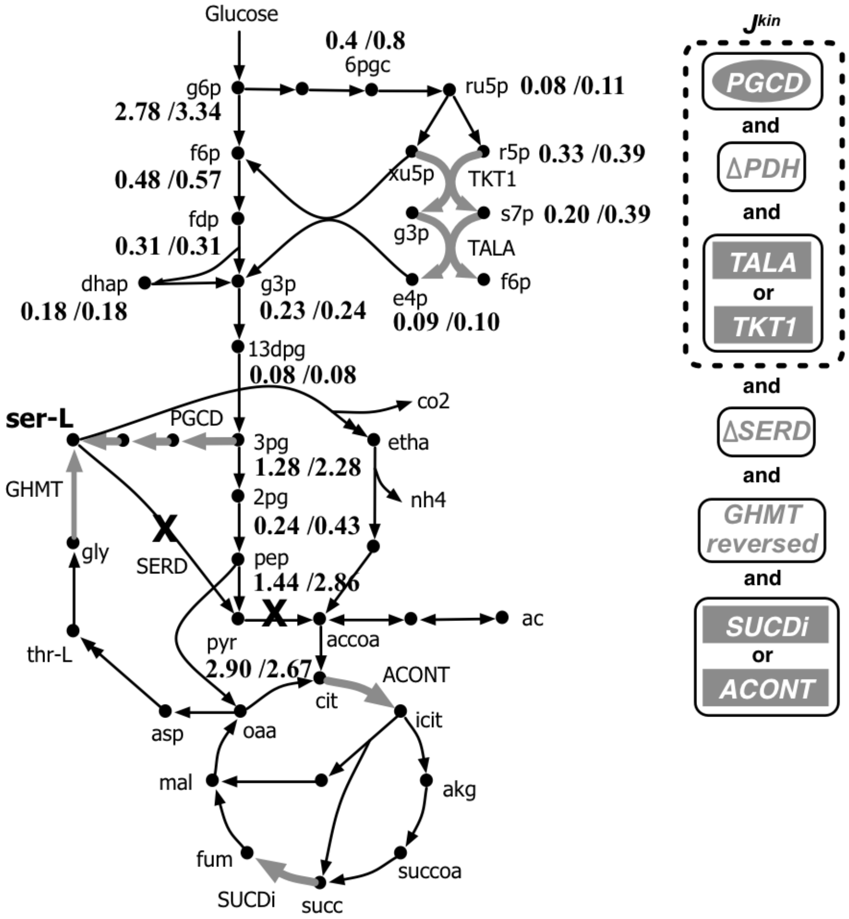

Figure S2: The concentration of metabolites (in mM) in the reference and overproduction phenotype for the overproduction of L-serine in *E. coli*. Metabolite concentrations of the reference phenotype (values on the right) are obtained by solving the system of ODEs for the kinetic model proposed in Chassagnole *et al*[1]. Metabolite concentrations of the engineered strain (values on the left) are obtained by solving for the FORCE sets.

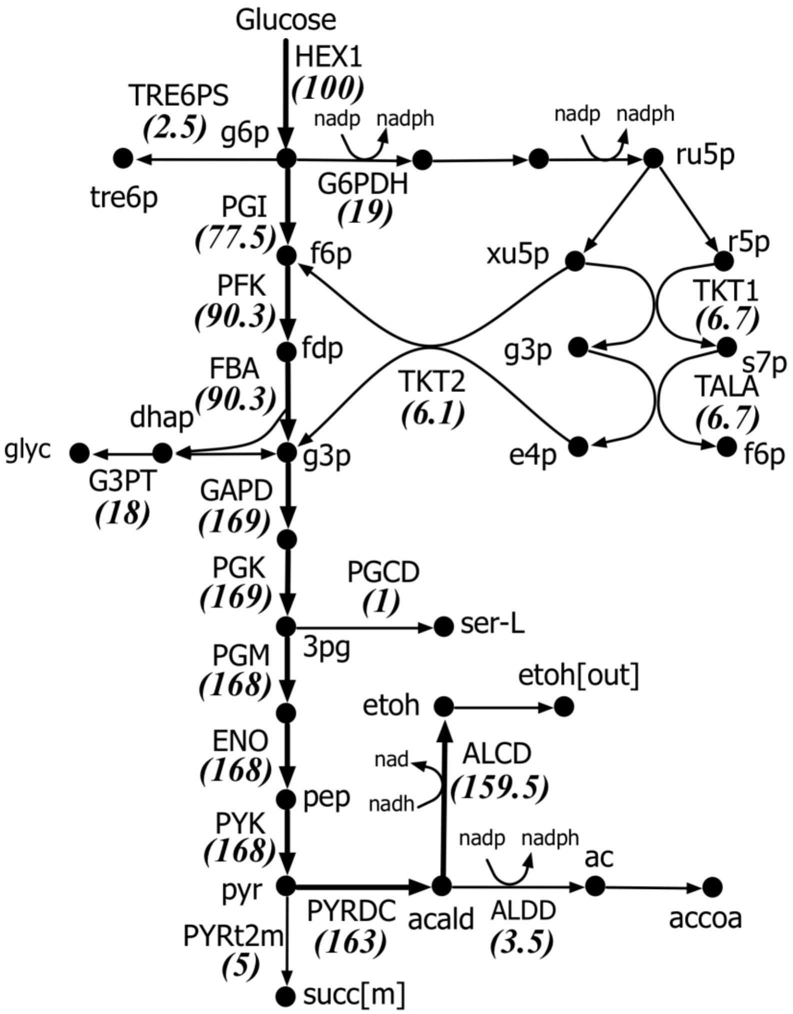

Figure S3: The steady-state distribution of fluxes in the central carbon metabolism of *S. cerevisiae* obtained after solving the system of ODEs for the kinetic model proposed in van Eunen*et al*[3]. Values in brackets indicate the flux in mmol $gDW^{-1}hr^{-1}$ per 100 mmol $gDW^{-1}hr^{-1}$ of glucose uptake.

Comparison of the flux ranges of key central metabolic reactions between the reference phenotype and the overproducing phenotype (for both k-OptForce and regular OptForce) for L-serine overproduction in *E. coli*:

Reaction	*Reference phenotype flux ranges (inmmol gDW^{-1}hr^{-1})*		*Overproducing phenotype flux range (k-OptForce) (inmmol gDW^{-1}hr^{-1})*		*Overproducing phenotype flux range (regular OptForce) (inmmol gDW^{-1}hr^{-1})*	
	Min flux	*Max flux*	*Min flux*	*Max flux*	*Min flux*	*Max flux*
GLCptspp	100	100	100	100	100	100
PGI	35.75	35.75	37.98	98.13	-35.96	99.81
PFK	73.35	73.35	0	292.46	0	292.46
FBA	73.34	73.34	0	102.22	-55.30	227.97
TPI	72.29	72.29	50.38	98.26	-25.73	98.82
GAPD	161.64	161.64	148.88	196.76	72.77	197.32
PGK	161.64	161.64	148.88	196.76	72.77	197.32
PGM	153.26	153.26	0	61.10	-108.85	61.81
ENO	153.26	153.26	0	61.10	-108.85	61.81
PYK	19.27	19.27	0	47.50	0	204.67
G6PDH	63.40	63.40	0	61.83	0	135.77
GND	63.37	63.37	1.68	22.72	0	22.72
RPE	37.74	37.74	0.39	14.42	-31.03	14.42
RPI	25.51	25.51	1.27	15.29	0.49	31.00
TKT1	20.86	20.86	0.39	7.40	-15.32	7.40
TALA	20.72	20.72	0.36	7.37	-15.35	7.37
TKT2	16.88	16.88	0	7.01	-15.71	7.01
EDA	0.03	0.03	0	47.88	0	121.18
EDD	0.03	0.03	0	47.88	0	121.18
PGMT	0.86	0.86	0.19	107.21	0.19	199.27
PDH	93.28	93.28	0	14.03	0	15.15
PPC	27.00	27.00	0	118.68	0	203.52
GLGC	0.77	0.77	0.15	7.05	0.15	1000
ACK	-96.15	476.47	-13.47	199.64	-14.59	199.64
G3PD	1.05	1.05	0.16	3.95	0.16	132.88
PGCD	9.38	9.38	153.82	212.23	153.82	263.49
PSERT	9.38	9.38	153.82	212.23	153.82	263.49
PSP	9.38	9.38	153.82	212.23	153.82	263.49
DDPA	3.84	3.84	0.36	2.11	0.36	2.88
GHMT	-51.53	6.76	-26.76	9.60	-26.76	12.92
PRPPS	5.02	5.02	0	11.29	0	199.96
SERD_L	0	58.28	0	41.04	0	81.87
CS	4.78	60.35	1.05	200.13	1.05	200.13
ACONT	4.78	62.11	1.05	52.02	1.05	55.29
ICDH	4.78	61.37	1.05	15.07	1.05	16.19
AKDGH	0	60.17	0	14.03	0	15.15
SUCOAS	-1000	60.06	-1000	68.90	-1000	68.95
SUCD	0	62.87	0	50.98	0	54.25
FUM	-9.74	69.81	-6.04	84.65	-6.60	85.10
MDH	-374.62	66.99	-243.60	87.29	-243.60	90.94
ICL	0	63.98	0	50.97	0	54.25
MALS	0	62.98	0	50.97	0	54.25
GLUSY	0	217.84	0	199.08	0	199.08
ASPTA	12.48	239.40	2.74	82.37	2.74	82.37
THRA	0	112.67	0	55.60	0	55.60
ACCOAC	9.59	527.43	2.10	2.32	2.10	201.18

Comparison of the flux ranges of key central metabolic reactions between the reference phenotype and the overproducing phenotype (for both k-OptForce and regular OptForce) for TAL overproduction in *S. cerevisiae*:

Reaction	*Reference phenotype flux range (inmmol gDW^{-1}hr^{-1})*		*Overproducing phenotype flux range (k-OptForce) (inmmol gDW^{-1}hr^{-1})*		*Overproducing phenotype flux range (regular OptForce) (inmmol gDW^{-1}hr^{-1})*	
	Min flux	*Max flux*	*Min flux*	*Max flux*	*Min flux*	*Max flux*
GLCt	100	100	100	100	100	100
HEX1	100	100	95.84	100	95.84	100
PGI	77.56	77.56	91.02	97.18	91.02	97.18
PFK	90.31	90.31	0	99.26	0	99.26
FBA	90.31	90.31	0	98.87	0	98.87
TPI	71.30	74.78	91.77	96.86	91.77	96.86
GAPD	168.58	168.58	191.83	197.65	191.83	197.65
PGK	168.58	168.58	191.83	197.65	191.83	197.65
PGM	167.69	167.69	191.83	197.65	191.83	197.65
ENO	167.69	167.69	191.83	197.65	191.83	197.65
PYK	167.55	167.55	191.68	197.50	191.68	197.50
PYRDC	163.03	163.03	155.61	179.90	155.61	179.90
ALCD	159.69	159.69	86.00	142.85	86.00	142.85
ALDD	3.53	3.53	66.53	68.91	66.53	68.91
ACS	0.47	126.14	69.95	106.24	69.95	106.24
PC	0	167.96	30.10	38.23	30.10	38.23
ACCOAC	0.36	35.32	46.65	48.05	46.65	48.05
FAS80	0	9.81	0	0.40	0	0.40
2PS	0	9.03	23.27	23.56	23.27	23.56
G6PDH	18.99	18.99	0	1.70	0	1.70
GND	18.99	18.99	0	1.70	0	1.70
RPE	11.65	12.39	-0.45	1.05	-0.45	1.05
RPI	7.33	7.33	-0.45	1.05	-0.45	1.05
TKT1	6.67	6.74	-0.18	0.56	-0.18	0.56
TALA	6.65	6.66	0	0.56	0	0.56
TKT2	6.01	6.07	-0.47	0.49	-0.47	0.49
G3PT	18.33	18.33	0	6.79	0	6.79
PYRt2m	4.74	4.74	-4.21	7.64	-4.21	7.64
PGCD	0.89	0.89	0	2.08	0	2.08
GHMT	-0.82	16.48	-0.45	2.17	-0.45	2.17
CSm	0	26.42	0	1.03	0	1.03
CSp	0	26.65	0	1.01	0	1.01
ACONT	0.93	27.18	0.29	1.04	0.29	1.04
ICDH	0	19.63	0	1.00	0	1.00
ASPTA	-258.43	81.88	19.66	36.00	19.66	36.00
THRA	0	33.30	0	34.59	0	34.59
TRE6PS	2.45	2.45	0.01	1.70	0.01	1.70

References:

1. Chassagnole C, Noisommit-Rizzi N, Schmid JW, Mauch K, Reuss M (2002) Dynamic modeling of the central carbon metabolism of Escherichia coli. Biotechnol Bioeng 79: 53-73.

2. Feist AM, Henry CS, Reed JL, Krummenacker M, Joyce AR, et al. (2007) A genome-scale metabolic reconstruction for Escherichia coli K-12 MG1655 that accounts for 1260 ORFs and thermodynamic information. Mol Syst Biol 3: 121.

3. van Eunen K, Kiewiet JA, Westerhoff HV, Bakker BM (2012) Testing biochemistry revisited: how in vivo metabolism can be understood from in vitro enzyme kinetics. PLoS Comput Biol 8: e1002483.

4. Gombert AK, Moreira dos Santos M, Christensen B, Nielsen J (2001) Network identification and flux quantification in the central metabolism of Saccharomyces cerevisiae under different conditions of glucose repression. J Bacteriol 183: 1441-1451.
